# Efficient Simultaneous Detection of Metabolites Based on Electroenzymatic Assembly Strategy

**DOI:** 10.34133/bmef.0027

**Published:** 2023-09-19

**Authors:** Anran Zheng, Chao Li, Shengkai Xu, Zhen Guo, Chuanyu Li, Changsong Zhang, Jia Yao, Zhiqi Zhang, Jinze Li, Lutao Du, Shasha Zhao, Chuanxin Wang, Wei Zhang, Lianqun Zhou

**Affiliations:** ^1^ CAS Key Lab of Bio-Medical Diagnostics, Suzhou Institute of Biomedical Engineering and Technology, Chinese Academy of Sciences, Suzhou 215163, China.; ^2^ School of Biomedical Engineering (Suzhou), Division of Life Sciences and Medicine, University of Science and Technology of China, Hefei 230026, China.; ^3^ Suzhou Hospital, Affiliated Hospital of Medical School, Nanjing University, Suzhou 215153, Jiangsu Province, China.; ^4^ Suzhou CASENS Co. Ltd., Suzhou 215163, China.; ^5^ Ji Hua Laboratory, Foshan 528000, China.; ^6^ Department of Clinical Laboratory, The Second Hospital of Shandong University, Jinan 250033, Shandong, China.

## Abstract

*Objective and Impact Statement*: We describe an electroenzymatic mediator (EM) sensor based on an electroenzymatic assembly peak separation strategy, which can efficiently realize the simultaneous detection of 3 typical cardiovascular disease (CVD) metabolites in 5 μl of plasma under one test. This work has substantial implications toward improving the efficiency of chronic CVD assessment. *Introduction*: Monitoring CVD of metabolites is strongly associated with disease risk. Independent and time-consuming detection in hospitals is unfavorable for chronic CVD management. *Methods*: The EM was flexibly designed by the cross-linking of electron mediators and enzymes, and 3 EM layers with different characteristics were assembled on one electrode. Electrons were transferred under tunable potential; 3 metabolites were quantitatively detected by 3 peak currents that correlated with metabolite concentrations. *Results*: In this study, the EM sensor showed high sensitivity for the simultaneous detection of 3 metabolites with a lower limit of 0.01 mM. The linear correlation between the sensor and clinical was greater than 0.980 for 242 patients, and the consistency of risk assessment was 94.6%. *Conclusion*: Metabolites could be expanded by the EM, and the sensor could be a promising candidate as a home healthcare tool for CVD risk assessment.

## Introduction

Cardiovascular diseases (CVDs) are chronic diseases influenced by multiple metabolic factors that cause a serious social problem because of their high incidence and recurrence [1,2]. This situation has been improved through chronic disease management and risk assessment of CVD [3,4]. Metabolic factors are the predominant risk factors for CVD. The efficient detection of multiple metabolites will facilitate the risk assessment of CVD, which is important for chronic disease management and is the key to delaying the onset of CVD and reducing mortality [5,6]. Glucose (Glu) [7], lactate (Lac) [8], and cholesterol (Chol) [9–11] are 3 typical metabolites that are closely related to the risk assessment of chronic CVD, reflecting the metabolic level in the body and vascular risks. Their levels outside the normal range have different but related effects on blood vessels; these effects increase the risk of CVD. In clinical practice, different instruments and blood collection tubes are required for different metabolite detections, which are time-consuming and require professional operators. This takes up a lot of medical resources and causes a huge medical burden [12]. Therefore, the efficient simultaneous detection of multiple metabolites from a single sample on a single chip is urgently required to efficiently monitor the risk of CVD. The development of a sensor that detects multiple metabolites simultaneously will substantially improve the detection efficiency and reduce medical stress.

Significant progress has been made in the development of multiple indicator simultaneous detection biosensors, and a variety of techniques such as fluorometry [13,14], electrochemistry [15–17], surface plasmon resonance [18], and optical sensors [19] have been reported. Electrochemical methods for easily integrated testing are superior to other methods in terms of time and cost, with the added advantage of allowing simultaneous detection. Because of the challenges of multienzyme immobilization, most sensors reduce the use of enzymes, and simultaneous detection has mainly been achieved via multiple chips, multiple channels, or multiple labels. In addition, the realization of the simultaneous detection of some sensors was based on the modification of novel materials, which was associated with more complex experimental manipulations, thus reducing the involvement of enzymes and limiting specificity for clinical diagnosis.

The advantages of electroenzymatic mediators (EMs) provide extensive potential for innovations in the field of electrochemical biosensors, such as increasing the specificity of detection or amplifying the current signal [20]. Their in-depth research still has the potential for innovation. Different EMs exhibit specific redox potentials and enzyme affinities. A potential shift can be achieved on the basis of the EM characteristics. Methylene blue (MB) has a low redox potential and unique electrochemical properties [21], whereas ferrocene (Fc) exhibits biostability and low toxicity; these compounds are widely used in biosensors and biomedicine. Coenzyme flavin mononucleotide is a flavoprotein that catalyzes redox reactions [22]. These are excellent EM with different redox potentials. Oxidase and horseradish peroxidase (HRP) are easily coupled to other molecules and may be immobilized on the surface of electrodes [23]. Graphene has been extensively used in electrochemical biosensors owing to its high carrier mobility and large specific surface area [24]. Graphene-modified materials are often utilized in biological microelectrochemical systems owing to their high conductivities and excellent biocompatibilities. These EM and nanometer materials have their own excellent properties; can we use them creatively to realize the simultaneous detection of multiple metabolites based on one sensor?

Here, we report a biosensor based on an electroenzymatic assembly transduction strategy for the simultaneous detection of 3 metabolites. Three EM layers were assembled on electrode surface for a potential shift. The EM peak separation (EMPS) method based on tunable potential was applied to peak separation under one test. Metabolites could be quantitatively and simultaneously detected using peak currents. The lower limit of sensitivity for detection was 0.01 mM, and the detection time was less than 30 s. In addition, the difference in detection between the phosphate-buffered saline (PBS) solution and plasma was corrected. The EM sensor was used to simultaneously detect Glu, Lac, and Chol in 242 clinical samples, and the linear correlations between the EM sensor and the laboratory results was greater than 0.980. The indicators could be readily extended by changing a suitable mediator based on the EMPS method, and the EM sensor had important practical value in clinical practice owing to good agreement and efficiency.

## Results

### Analysis of the EM sensor in feasibility and interference experiments

**Fig. 1.**
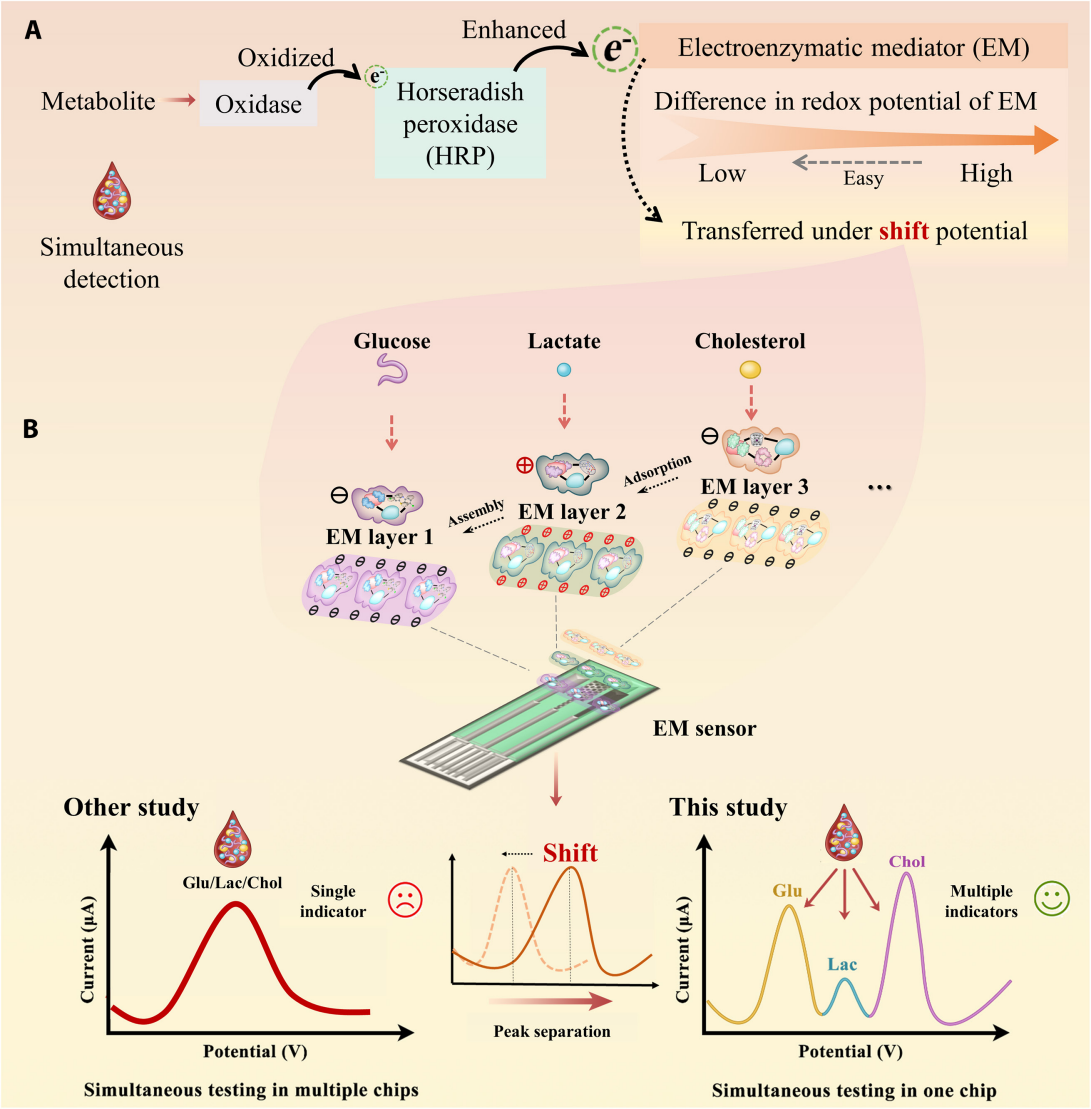
Principle of simultaneous detection by the EM metabolite sensor. (A) Mechanisms of electronic transmission. (B) Assembling 3 EM layers on the electrode and peak appeared at different shift potentials.

The cross-linking design and characterization process of the EM sensor are shown in the Supplementary Materials (Figs. S1 and S2 and Table S1). Further studies on the dielectric pathway were performed after determining the completion of the cross-linking of each EM layer. The principle of the EMPS method for the detection of metabolites Glu, Lac, and Chol is shown in Fig. 1. In this method, a recognition layer composed of specific oxidase enzymes is used, which allows for the specific recognition and oxidation of the targeted metabolite, leading to the generation of electrons. An amplification layer consisting of HRP is utilized to enhance the detected current signal. Furthermore, a differentiated layer incorporating different EMs serves the purpose of mediating the generated current to be detected at a specific potential, which is directly associated with the redox potential of the EMs. Ultimately, this approach enables the simultaneous detection of multiple metabolites. The EMPS method in this study achieves the simultaneous detection of different metabolites by shiftable potential on the same curve, it is based on the principle of detection of classical electrochemistry enzyme sensors [25,26], which detects the 3 current generated by redox reactions in one sample.

The repeatability experimental results include the impedance repeatability testing and metabolite testing under electroenzyme layer modification of the same and different batches of sensors. These results demonstrate excellent sensor repeatability and were shown in Tables S2 and S3 and Fig. S3.

**Fig. 2.**
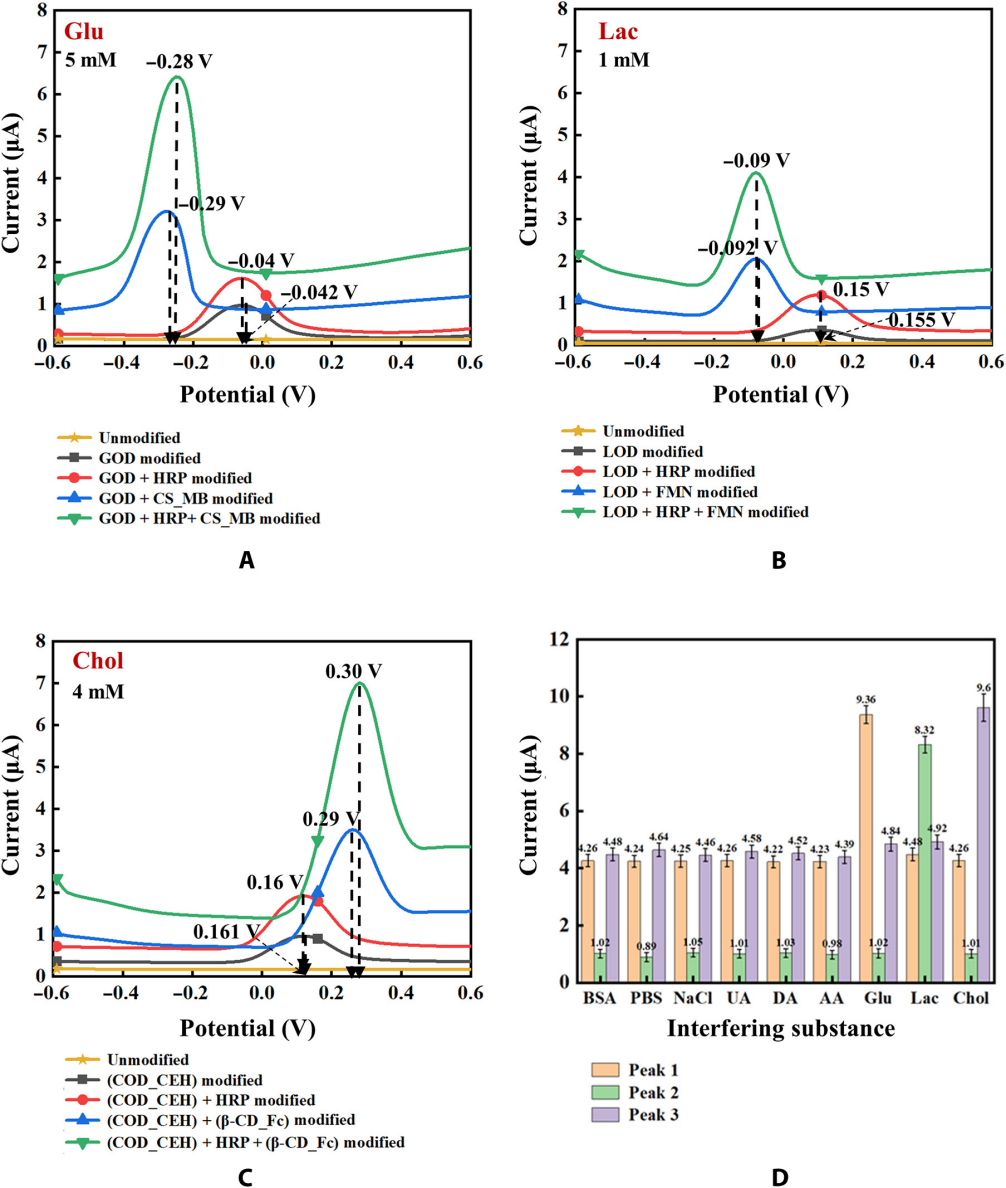
DPV test results of feasibility experiment of potential shift and anti-interference experiment in PBS. (A) Different modification test with 5 mM Glu. (B) Different test with 1 mM Lac. (C) Different test with 4 mM Chol. (D) Results of interference experiment. β-CD, β-cyclodextrin.

The results of the feasibility and interference analysis and comparison are shown in Fig. 2A to D. In general, electron transfer in living organisms occurs from low redox potentials to high redox potentials, but the direction may vary depending on enzyme specificity and inhibition [27]. Without modifications, the differential pulse voltammetry (DPV) test showed no peaks. Upon oxidase modification, the process of metabolite oxidation is catalyzed, and flavine adenosine dinucleotide (FAD) in the enzyme active center undergoes a redox reaction, resulting in the transfer of electrons. During the DPV test, peaks appeared at −0.04, 0.155, and 0.161 V, respectively. These potentials were larger than the standard redox potential of FAD, which may be related to the Ag/AgCl reference electrode used and the protection of the enzyme afforded by HRP during the reaction with H_2_O_2_ [28].

During the electroenzymatic assembly, the conformation of the enzyme may change. Electron transfer between the enzyme active site and electrode can occur with different efficiencies. The peak potentials detected were −0.29, −0.092, and 0.29 V. The current signals of the peaks further increased, and the tests became more stable after the addition of HRP. Notably, MB can improve electrode performance through electropolymerization and can be adsorbed by Chitosan (CS) with high selectivity [29–31]. The large number of amino and carboxyl groups on the CS surface is critical in the adsorption of MB and the connection of Glu oxidase (GOD) in EM layer 1. The electrochemical properties of MB indicate the lower oxidation potential; the same is true for the other 2 EM layers. Flavin mononucleotide enhances and assists in the oxidation of Lac by Lac oxidase (LOD). Fc had lipophilicity, and the host–guest structure formed with β-cyclodextrin could entrap Chol oxidase (COD) more closely, making the recognition of Chol more specific, and the peak appeared at the potential of Fc (0.3V). The anti-interference test results (Fig. 2D) demonstrated good sensitivity of the EM metabolite sensor for the detection of Glu, Lac, and Chol.

### Gradient test of single metabolite under single EM layer modification in PBS

**Fig. 3.**
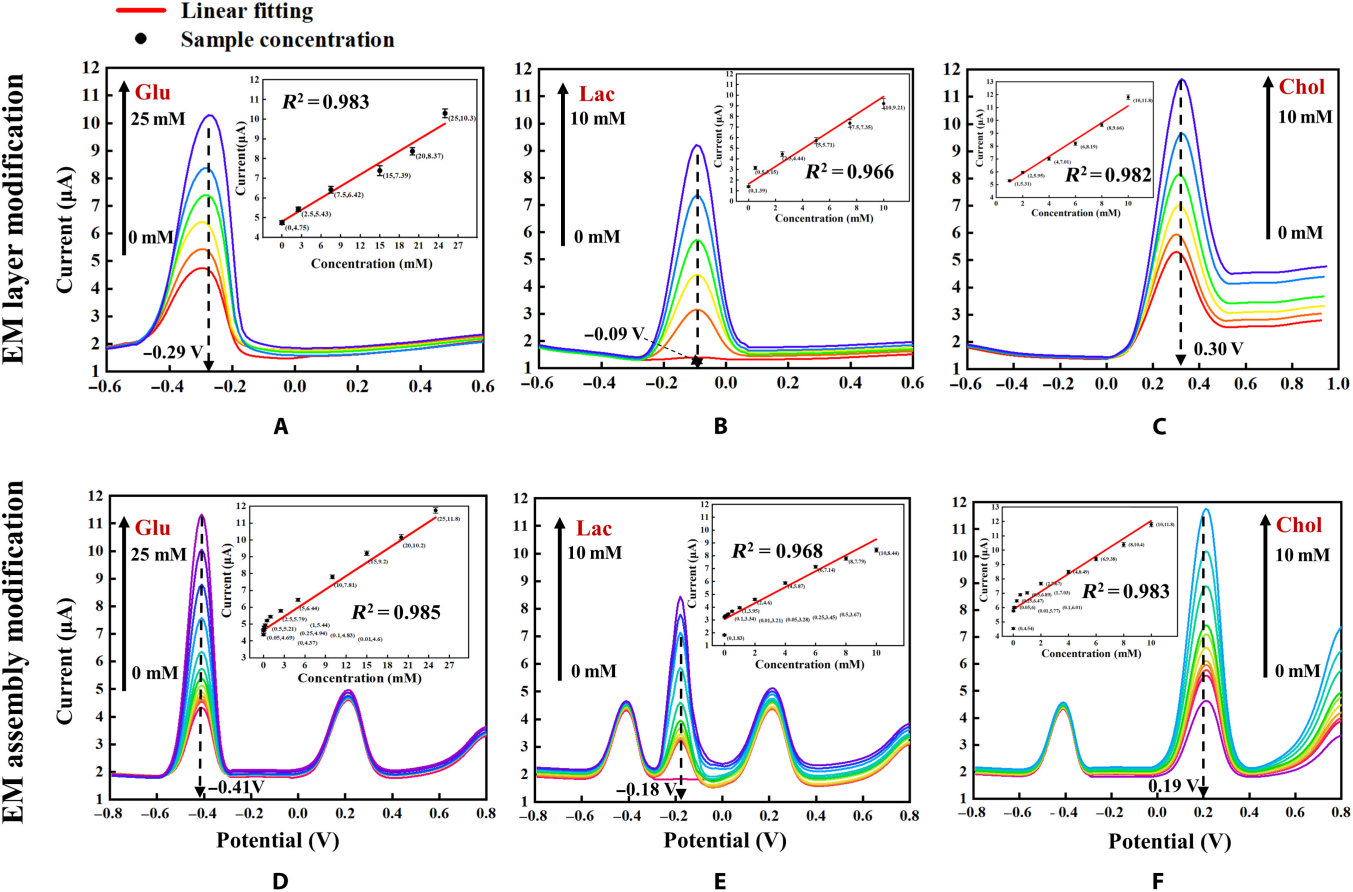
Three metabolites gradient tested in PBS. (A) Glu testing under EM layer 1 modifications. (B) Lac testing under EM layer 2 modifications. (C) Chol testing under EM layer 3 modifications. (D to F) Glu, Lac, and Chol gradient testing under EM assembly modification.

The results of the single-metabolite gradient tests upon modification of the optimal EM assembly are shown in Fig. 3A to C. The detected peak potentials were −0.29, −0.09, and 0.30 V, respectively. The concentration gradients of Glu (0 to 25 mM), Lac (0 to 10 mM), and Chol (0 to 10 mM) correlated well with the respective peak currents, with linear correlations (*R*^2^) of 0.983, 0.966, and 0.982, respectively. The detection concentration of 3 metabolites was determined on the basis of clinical requirements, and the setting of detection accuracy as well as the upper and lower limits of the sensor gradient experiment was guided by the clinical detection range. The peak did not appear at −0.09 V in the absence of Lac. The results of single-metabolite gradient testing under assembly modification are shown in Fig. 3D to F. The concentration gradients of Glu (0 to 25 mM), Lac (0 to 10 mM), and Chol (0 to 10 mM) correlated well with their respective peak currents. Negative shifts in the peak potential were observed, but each metabolite was still linearly related to the peak current for each EM layer, and the consistency was good. Their distinguishable potentials shifted because of the competition and promotion of redox reactions in the same system. Similarly, the peak did not appear at −0.18 V in the absence of Lac.

### Gradient test of metabolites mixture under 3 EM layers modification in PBS and plasma

**Fig. 4.**
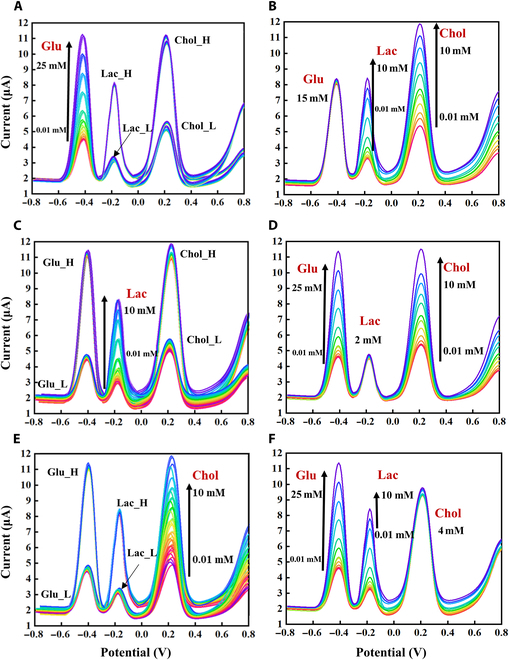
Gradient test results of mixed sample in PBS background. (A) Glu gradient testing. (B) Lactic acid and Chol gradient tests. (C) Lac gradient test. (D) Glu and Chol gradient tests. (E) Chol gradient testing. (F) Glu and Lac gradient tests.

**Fig. 5.**
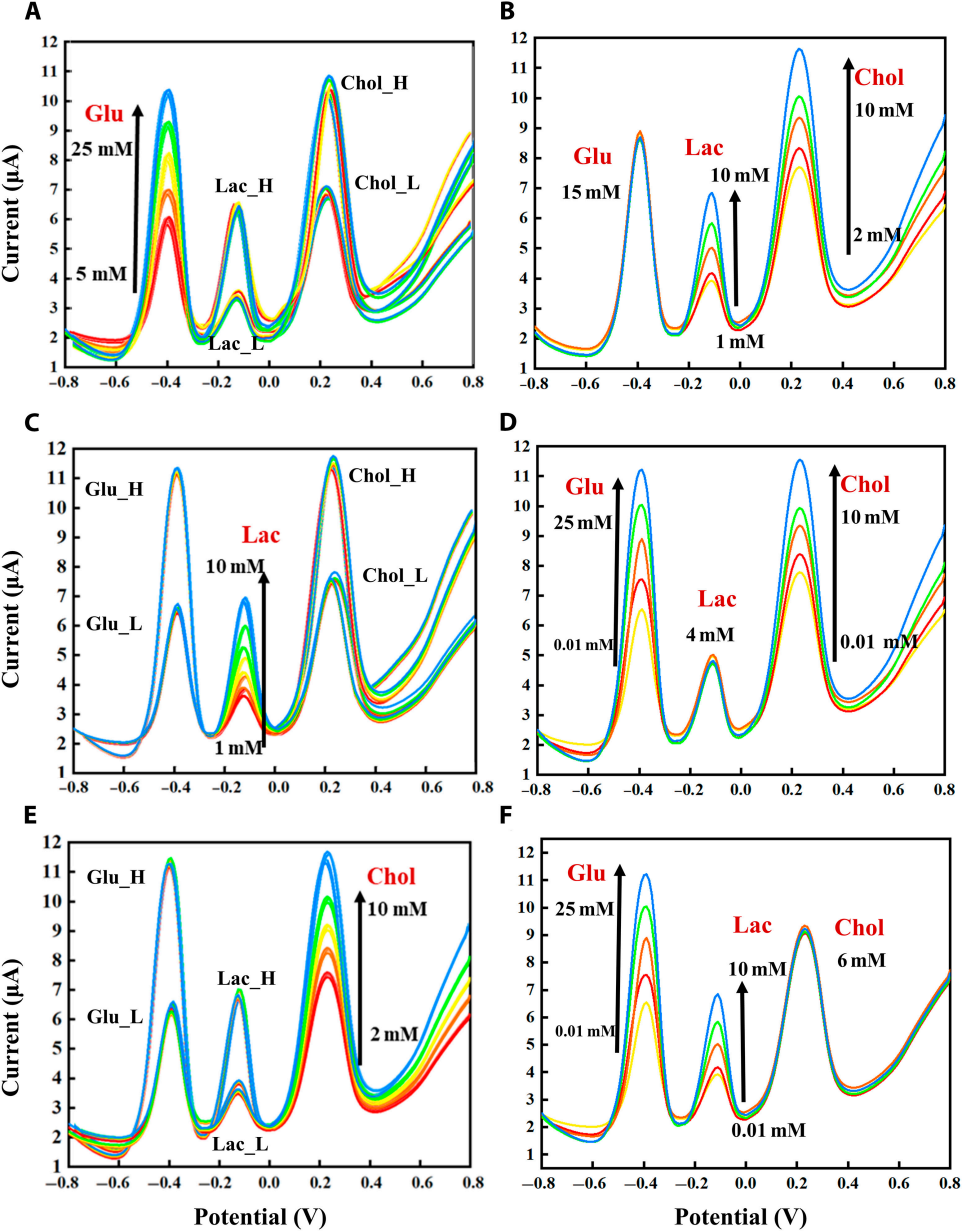
Gradient test results of mixed sample in plasma background. (A) Glu gradient testing. (B) Lactic acid and Chol gradient tests. (C) Lac gradient test. (D) Glu and Chol gradient tests. (E) Chol gradient testing. (F) Glu and Lac gradient tests.

The results for the under 3 EM layers assembly modifications of the 3 metabolites in PBS are shown in Fig. 4. The results of the multiple gradient tests proved the good sensitivity and calibration of the 3 dielectric pathways for the detection of Glu, Lac, and Chol. In addition, the results after the assembly modifications under 3 EM layers of the 3 metabolites in plasma are shown in Fig. 5.

The detailed descriptions of the detected metabolite concentrations in Figs. 4 and 5 can be found in Section S1.4. Moreover, it is worth noting that plasma, being rich in proteins, bioelectrolytes, and other components [32], introduces additional challenges during the gradient test compared to PBS. Specifically, the intracellular metabolite metabolism has a greater impact on the plasma-based gradient test. Consequently, calibration was performed to account for the varying backgrounds encountered.

### Linear regression analysis and calibration in PBS and plasma

**Fig. 6.**
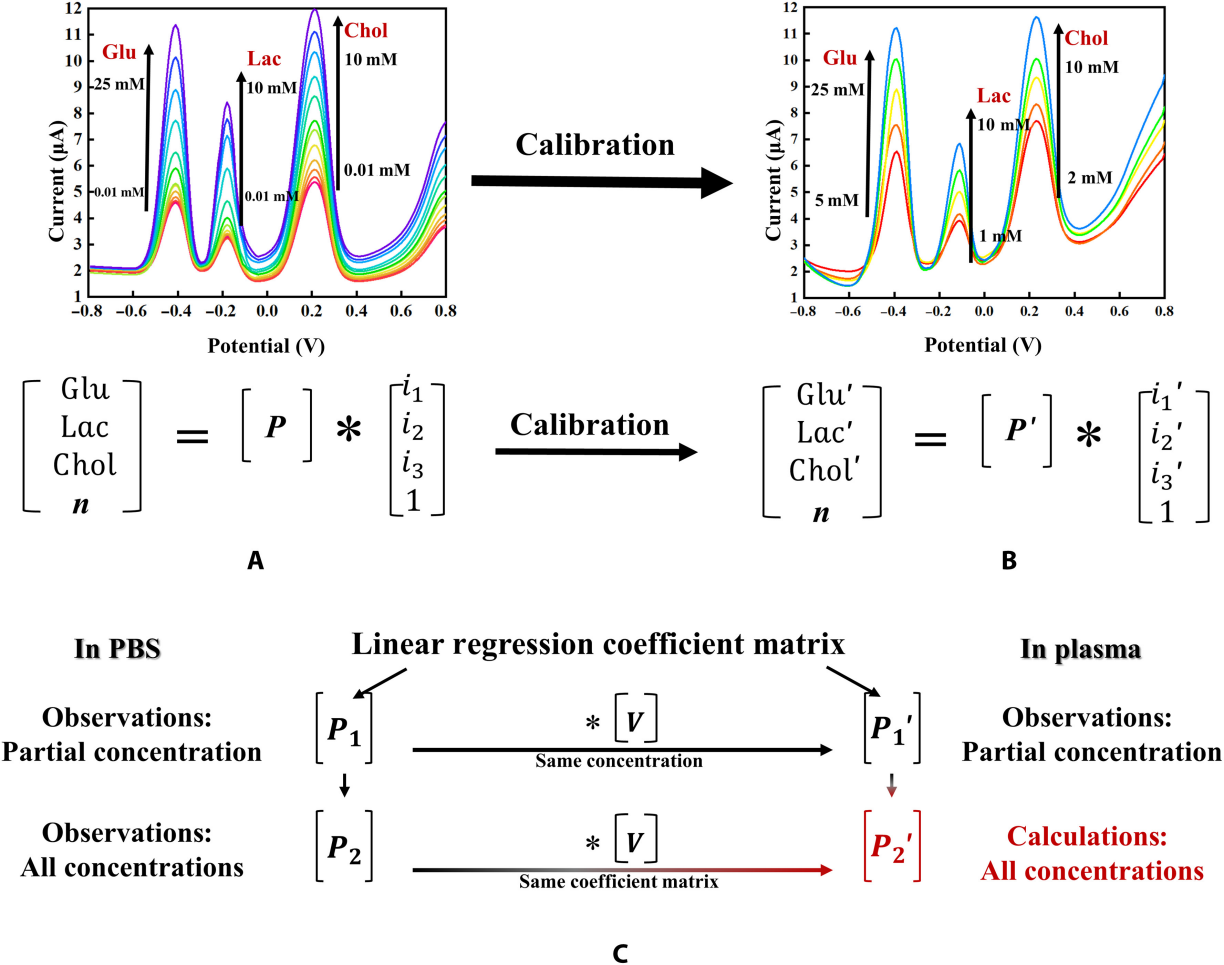
Background calibration from PBS to plasma. (A) Test results and equation of full gradient experiments in PBS. (B) Test results and equation of partial gradient experiments in plasma. (C) Linear regression analysis.

Linear regression [33] was used to analyze the metabolite peak current detection data in PBS and plasma. The details are provided in the Supplementary Materials (Tables S4 and S5). The matrix [P_1_] of the partial gradient test under PBS, matrix [P_2_] of the full gradient test, and matrix [P_1_′] of the partial gradient test under plasma were obtained. The full gradient matrix [P_2_′], corrected by the coefficient matrix, was used to correct the test results for Glu and Chol in the plasma [33]. The calculation processes are shown in Fig. 6C and Eqs. S1 to S19. The concentration of metabolites in the sample was calculated using Eq. S20.

### Clinical validation and risk assessment of the EM sensor

**Fig. 7.**
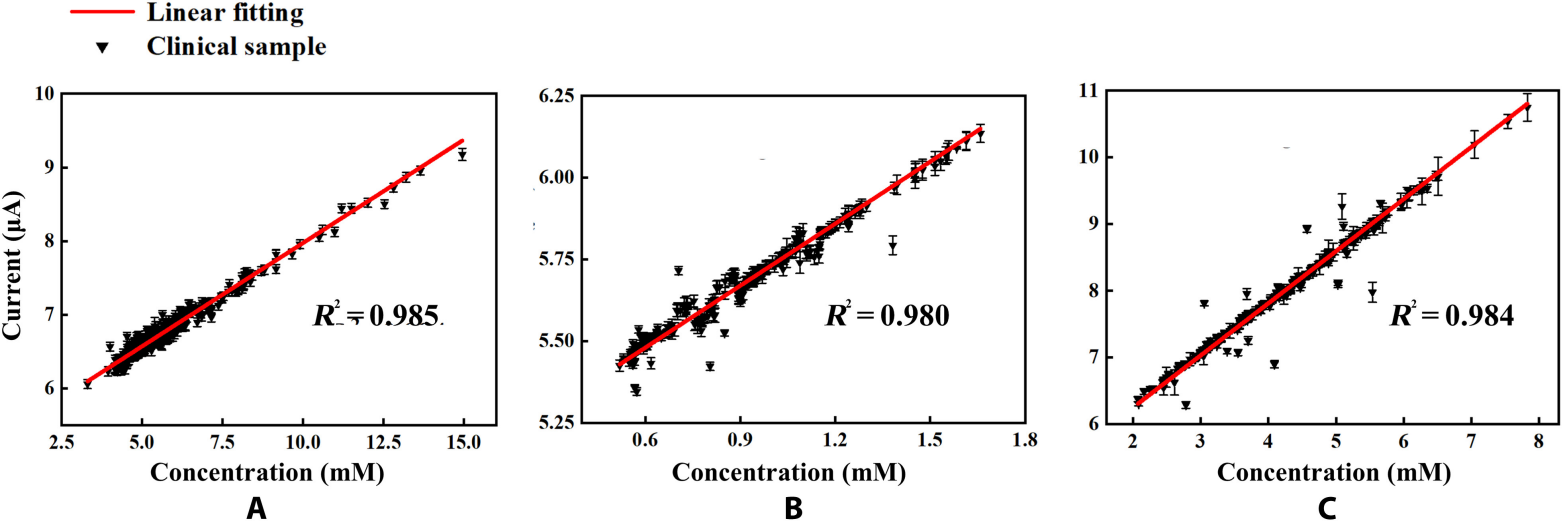
Results of linear fitting between the EM sensor and the clinical instrument. (A) Result of Glu clinical test. (B) Result of Lac clinical test. (C) Result of Chol clinical test.

On the basis of the calibrated matrix equation, clinical validation of the blood samples collected from 242 patients in different departments was performed. DPV curves were recorded, and the levels of each metabolite were calculated and validated using a Siemens automatic biochemical analyzer. Figure 7A to C shows the results for 242 patients. The linear correlations between the EM sensor and Siemens automatic biochemical analyzer for Glu, Chol, and Lac detection were 0.985, 0.980, and 0.984, respectively. The sensor shows good consistency with the clinical instrument, and the data are shown in Table S6.

## Discussion

In most biological systems, recognizable reactions do not occur without the addition of enzymes or electron transporters [34]. As shown in Fig. 1, the oxidases containing GOD, LOD, and COD were used to specifically identify Glu, Lac, and Chol, respectively. These oxidases contained the same FAD active center. HRP decomposes H_2_O_2_ that is produced by redox reactions, into water and oxygen, and was used to decrease test interference and amplify current signals. The EM was flexibly designed by cross-linking the electron mediator and enzyme, guiding the electron transfer at the shift potential. With the consideration of triple modification, a cross-linking adsorption approach was used to assemble the EM layer, primarily based on its diverse charged properties. The modification was executed in a sequential, and investigations have been conducted on both individual and mixed modes of modification. To enhance the stability of the determination, the EM layer modification solutions were subjected to separate cross-linking, enabling the electrons to be recognized and transferred in a directed manner. This facilitated a further reduction in distance and improvement in efficiency. This is not only physical contact but also directional binding, which improves the efficiency of electron transfer and enhances the signal.The issue of potential diffuse back is taken into consideration through multilayer modification, thus directing our attention toward 2 key aspects of analysis. On the one hand, the stability was enhanced between functional molecules within each electroenzymatic layer using cross-linking through chemical bonds. On the other hand, layer-to-layer assembly is achieved via electrostatic charge adsorption. The 3 EM layers exhibited different electrical properties and were assembled via electrostatic adsorption. Both approaches transcend mere physical assembly, contributing to the assurance of test stability and repeatability to a certain extent. The electroenzymatic modification method presented in this paper demonstrates relative stability in plasma, exhibiting remarkable sensitivity and specificity. When a sample entered the EM metabolite sensor, Glu, Lac, and Chol were recognized specifically by oxidase. The current signals were amplified through HRP, and electron transfer was promoted under the shift potential of different EMs. The higher the sample concentration, the more electron transfer occurred, and the peak current is related to the sample concentration. Thus, the peaks of the 3 metabolites on one detection curve were separated, and simultaneous detection was achieved in one chip.

The feasibility and anti-interference test results demonstrated good sensitivity of the EM metabolite sensor for the detection of Glu, Lac, and Chol. In addition, the 3 metabolite concentrations had different weight relationships with the 3 peak currents in each gradient test. When the samples contained Glu, Lac, and Chol simultaneously, the combined gradient test results at high and low concentrations of the different substances were used to analyze the mutual interference between the tested substances. The results of multiple sets of gradient tests further proved the good sensitivity and correlation of the 3 dielectric pathways for the detection of Glu, Lac, and Chol.

In addition, plasma contains more proteins, bioelectrolytes, and other components. The gradient test based on plasma was more disturbed by intracellular metabolism than was the gradient test based on PBS. The fitting analysis results of the partial gradient in the plasma and the full gradient in the PBS show that the trend of gradient test in the plasma was consistent with the PBS experimental but the values were different. By multiplying the partial regression coefficient matrix [P_1_′] by the correction coefficient matrix [V], we obtained the full gradient regression coefficient matrix [P_2_′] under the plasma. Therefore, the results of Glu, Lac, and Chol test in the plasma could be more accurate by background correction and were demonstrated by the results of the clinical 242 patients.

The design, manufacturing, and performance optimization of sensors are all aimed at enhancing their practical value in the field of biomedical engineering. Thus, after conducting background calibration and verifying clinical samples, further investigation was conducted on the detection data of these clinical samples. Statistical analysis was performed on collected basic information, including blood pressure, age, and disease history of the inpatients. This information was combined with the results of biochemical indicators detected by EM sensors. Through single-factor analysis and multifactor analysis, a CVD risk assessment model and its classification criteria were established. The results were presented in the form of a risk output graph, which visually represents the assessed risk level, facilitating better self-management of chronic diseases for the individuals being tested.

**Fig. 8.**
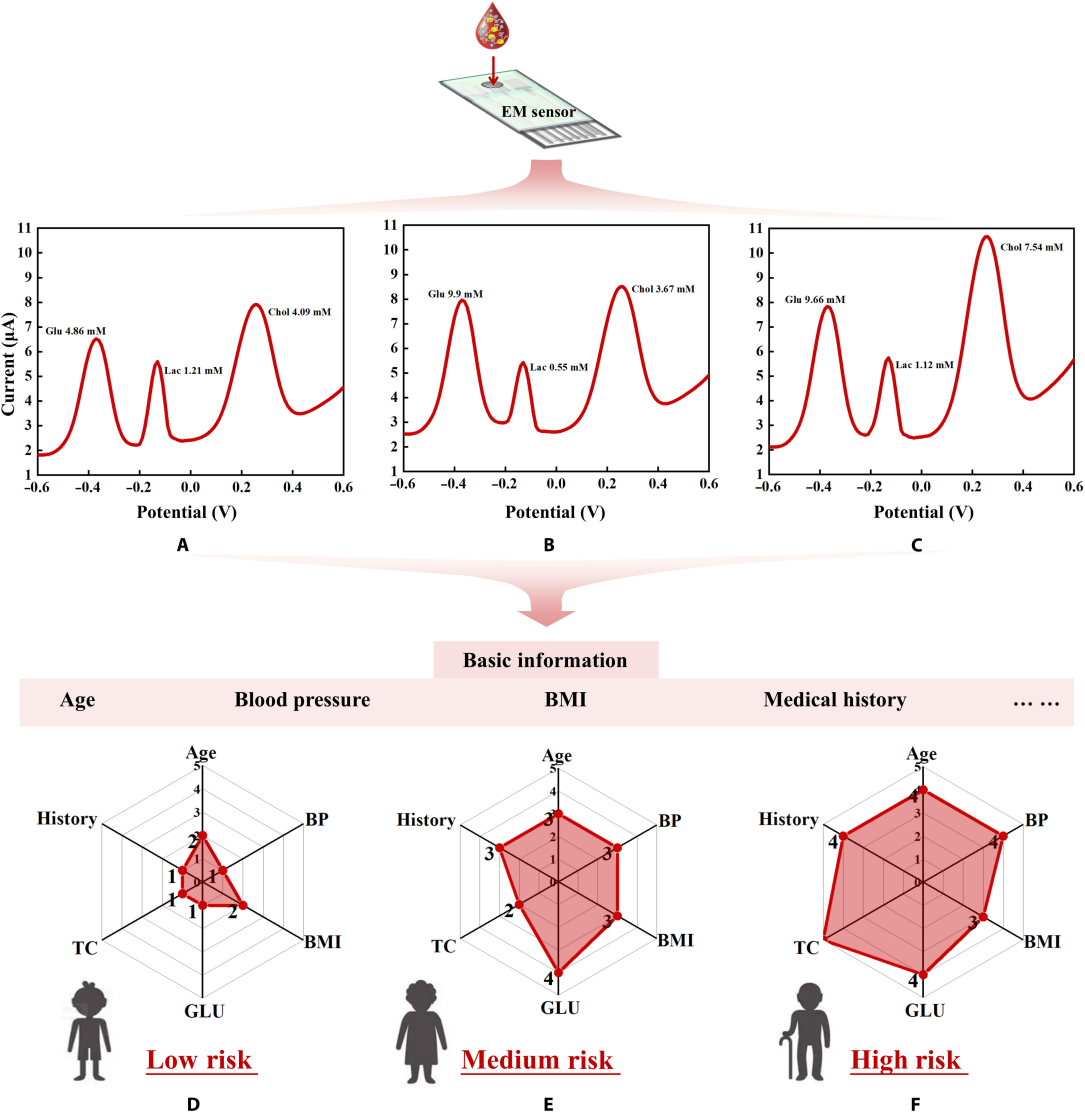
Test results and risk assessment of the EM sensor in clinical. (A to C) DPV tests of clinical samples 1 to 3. (D to F) Evaluation radar map of clinical samples 1 to 3. BMI, body mass index. BP, blood pressure; TC, total cholesterol.

An evaluation of the risk assessment model (Tables S12 and S13) based on the EM metabolite sensor and analysis is described in the Supplementary Materials (Tables S7 to S11). The sensor test and model evaluation results of the 3 representative patients are shown in Fig. 8. For sample 1, Glu, Lac, and Chol were at normal levels and belonged to the low-risk population. Sample 2 had high Glu, no diabetes, grade II hypertension, and a body mass index of 25.6 and belonged to the medium-risk category. Sample 3 was older and had high Glu and Chol levels. The patient also had a history of hypertension and diabetes and was at a high risk. The sensor detection results, combined with physiological parameters and personal conditions, were used to comprehensively analyze the risk level for CVD and output a radar map, which intuitively showed the disease risk. The consistency with the clinical diagnosis was 94.6% (Tables S14 to S17). This sensor is expected to have important clinical applications in home health care.

CVD are a major threat to global health and account for a large share of medical resources. This study proposes an EM sensor to simultaneously detect triple metabolites based on the EMPS method and establishes a model to evaluate the risk of CVD by combining basic information. This method includes oxidase for specific recognition, HRP for amplification, and EM layer for shift potential. The simultaneous detection of Glu, Lac, and Chol in the blood on one sensor is based on the discrimination of the peak potential and the current correlation with concentration. The background differences between the PBS and plasma were calibrated. The ranges of Glu, Lac, and Chol detection were 0.01 to 25 mM, 0.01 to 10 mM, and 0.01 to 10 mM, respectively. The concentration of each metabolite in the mixture was calculated using the following equation (Eq. S20). For the 242 clinical patients, the linear fitting correlations of Glu, Lac, and Chol were 0.985, 0.980, and 0.984, respectively. The EMPS method enabled the detection of 3 metabolite indicators; however, it was not limited to 3 indicators. Therefore, this sensor is expected to have broad application value in the fields of community hospitals and home medical care for CVD.

## Materials and Method

### Reagents and equipment

PBS (pH 7.0), NaCl solution, and glutaraldehyde were purchased from Lianshuo Biological Co. Ltd. (Shanghai, China). CS, bovine serum albumin, l-lactic acid, Glu, GOD, LOD, HRP, COD, Chol esterase, MB, flavin mononucleotide, β-cyclodextrin, and Fc were purchased from Sigma-Aldrich. A CHI660E electrochemical workstation (CH Instrument Co., Shanghai, China) was used in the electrochemical experiment. A Malvern Zetasizer Nano ZS90 (Malvern Instruments Ltd., United Kingdom) was used for the zeta potential and size analyses. The SPSS 20.0 statistical software was used SPSS analysis.

### Fabrication and modification of the EM sensor

The EM sensor was designed to include the independent reference, working, and counter electrodes. The injection port was located at the center. The fabrication process has been described in our previous work [35]. Before modification, the electrode in PBS (pH 7.0) was scanned by cyclic voltammetry at a sweep rate of 50 mV/s in the range of −0.2 to 0.6 V until stable. Optimization experiments for the preparation and cross-linking of different EM were then performed for better detection. EM modification solutions containing EM with different potentials, oxidase, and HRP were prepared and cross-linked for 4 h at 37 °C. In the order of EM layers 1 to 3, 2 μl of the EM layer solution was dropped onto the electrode surface and dried. The details of the preparation and optimization processes are provided in the Supplementary Materials. The hydrophilic material and electrode substrate were bonded using bonding materials, and the EM sensor was encapsulated for detection.

### Feasibility and interference experiment of the EM sensor

Zeta potential and particle size testing were performed using ZS90 to determine the extent of adsorption assembly and cross-linking of the EM solution. In the feasibility experiment, the same concentrations of Glu, Lac, and Chol solutions were applied in a single metabolite test with different modifications. The concentrations of Glu, Lac, and Chol were 5, 1, and 4 mM, respectively. The specificity of the EMPS method was verified using interfering biomolecules, including 0.9% NaCl, 0.5% bovine serum albumin, 15 mM Glu, 10 mM Lac, 6 mM Chol, 1 mM ascorbic acid, 1 mM uric acid, and 1 mM dopamine. DPV curves were obtained using an electrochemical workstation.

### Metabolite gradient test in PBS and plasma

The same concentrations of Glu, Lac, and Chol solutions were applied in different tests with signal EM layer and mixed modifications. Metabolite gradient testing in PBS was performed with EM layer modification, and the Glu concentrations were 0.01, 0.05, 0.1, 0.25, 0.5, 1, 2.5, 5, 10, 15, 20, and 25 mM. The Lac concentrations were 0.01, 0.02, 0.05, 0.1, 0.25, 0.5, 1, 2, 4, 6, 8, and 10 mM, and the Chol concentrations were 0.01, 0.02, 0.05, 0.1, 0.25, 0.5, 1, 2, 4, 6, 8, and 10 mM.

Under the EM assembly modification, the PBS background experiment was repeated with the same concentration gradient. In the plasma background experiment, the concentrations of Glu were 5, 10, 15, 20, and 25 mM, the concentrations of Lac were 1, 2, 4, 6, and 10 mM, and the concentrations of Chol were 2, 4, 6, 8, and 10 mM. Each experiment was repeated thrice.

### Linear regression analysis and coefficient matrix correction

The SPSS software was used for linear regression analysis [36] of the first group of data (all data corresponding to the test gradient under the plasma background selected from the full set of gradient data tested in the PBS), second group of data (all data tested in the plasma), and third group of data (full gradient data tested in the PBS). The detailed calculation processes are presented in the Supplementary Materials.

### Clinical validation of metabolite simultaneous measurements

Clinical tests were performed to verify the performance of the sensor on clinical test samples. A total of 242 patients were recruited from different departments during this test. The test was performed on the EM sensor using plasma (5 μl) and repeated thrice for each patient. The peak currents of the DPV curves were recorded, the level of each metabolite was calculated, and the results were validated using laboratory equipment in the hospital.
