# Supplementary material for: Efficient Simultaneous Detection of Metabolites Based on Electroenzymatic Assembly Strategy
Source: BME Front. 2023 Sep 19;4:0027. doi: 10.34133/bmef.0027 (PMC10530654; doi:10.34133/bmef.0027)
Supplement: Supplementary 1 — Figs. S1 to S3 [file bmef.0027.f1.docx]

**Efficient simultaneous detection of metabolites based on electroenzymatic assembly strategy**

Anran Zheng ^a,b^, Chao Li ^b,e^, Shengkai Xu ^c^, Zhen Guo ^a,b,e^, Chuanyu Li ^a,b,d^, Changsong Zhang ^c^, Jia Yao ^a,b^, Zhiqi Zhang ^a,b^, Jinze Li ^a,b d^, Lutao Du ^f^, Shasha Zhao ^b^, Chuanxin Wang ^f,*^, Wei Zhang ^a,b,e,*^, Lianqun Zhou ^a,b,d,e,*^

a School of Biomedical Engineering (Suzhou), Division of Life Sciences and Medicine, University of Science and Technology of China, Hefei, 230026, China

b CAS Key Lab of Bio-Medical Diagnostics, Suzhou Institute of Biomedical Engineering and Technology, Chinese Academy of Sciences, Suzhou, 215163, China

c Department of Laboratory Medicine, The Affiliated Suzhou Science and Technology Town Hospital, Nanjing Medical University, Suzhou 215153, Jiangsu Province, China

d Suzhou CASENS Co., Ltd, Suzhou, 215163, China

e Ji Hua Laboratory, Foshan, 528000, China

f Department of Clinical Laboratory, The Second Hospital of Shandong University, 250033, Jinan, Shandong, China.

Corresponding author E-mail addresses: zhoulq@sibet.ac.cn (L. Zhou), zhangw@sibet.ac.cn (W. Zhang), [cxwang@sdu.edu.cn](mailto:cxwang@sdu.edu.cn) (C. Wang)

**Contents** *Page*

1. Electrochemical study of EM metabolite sensor……………………………….……..……………...........3
   1. Modification details of EM metabolite sensor……………………………….….……………….…..3
   2. Modification characterization of EM metabolite sensors………………………………….……….…3
   3. Analysis on the optimization of modification condition…………………………………….…..….…5
   4. Gradient test and calibration of EM metabolite sensor in different background…….………………..8
   5. Clinical validation data of EM metabolite sensor ……….……………..…………..……………......14
2. Evaluation study of SELF model based on EM metabolite sensor……………..……………............…...19
   1. Comparison and analysis of different model for CVDs risk assessment……………...…...........…...19
   2. Analysis on baseline data and establishment of SELF model………………………….…….…........20
   3. Clinical validation results and information……………………………………….……..………...….25
3. Language Editing Certificate …………………………………………………………......……….....…...31
4. Reference………..……………………………………………………………………….............….......…32

1. Electrochemical study of EM metabolite sensor

1.1 Modification details of EM metabolite sensor

During the prepare process, first, an HRP solution was prepared from 5 mg of HRP powder dissolved in 0.5 ml of PBS solution. MB powder (3.2 mg) was dissolved in 10 mL of PBS to prepare a 1 mM MB solution. A total of 2 mg of GOD and 0.5 mg of CS powder were dissolved in PBS buffer, incubated with MB solution and HRP solution at a ratio of 1:1:2, and mixed well by ultrasonication to prepare EM 1 solution. After 4 hours of cross-linking at 37 °C, two microliters of the mixture were dropped onto the electrode surface and dried. Second, 2 mg of LOD and 0.5 mg of FMN powder were dissolved in PBS buffer and ultrasonicated for 30 min. Five milligram of HRP powder dissolved in 0.5 ml of 2.5% glutaraldehyde in a 37 °C water bath for 2 h, incubated with above solution at ratio of 1:1 to prepare EM 2 solution. Cross-linking and drying under the same conditions. Third, 18.5 mg of cyclodextrin powder was dissolved in 30 µL of a 0.1 M NaOH (50 mg/mL) solution, and then the volume was brought to 1 mL. After stirring at 60°C for 2 hours, 3.75 mg of Fc powder was weighed and added in three equal amounts, and the resulting solution was pumped and filtered to form a β-CD-Fc solution. Two milligrams of COD, 3 mg of HRP and 2 mg of CEH powder were dissolved in PBS and ultrasonicated for 30 min, mixed with above solution at ratio of 1:1 to prepare EM 3 solution. Cross-linking and drying under the same conditions.

1.2 Modification characterization of EM metabolite sensors

After modification, the ZS90 was used as a test instrument for the characterization of the modification, and for each layer of the EM solution the zeta potential and particle size were tested, and the single substance contained in each EM layer was also tested under the same conditions.

**Tab. S1** Characterization results of electroenzymatic assembly performance

| Sample | Zeta potential (mV) | Size (nm) |
| --- | --- | --- |
| GOD | -26.9 | 70.49 |
| LOD | -3.78 | 27.7 |
| COD | -5.71 | 36.31 |
| CEH | -3.71 | 69.05 |
| HRP | 4.54 | 57.26 |
| GA | 4.46 | 39.53 |
| FMN | -4.26 | 57.88 |
| MB | -2.55 | 36.9 |
| FC | 4.58 | 51.12 |
| CS | 2.27 | 75.21 |
| β-CD | -6.24 | 193.7 |
| CS-MB | 1.6 | 191.1 |
| GOD+HRP | -27.5 | 171.8 |
| CS-MB+GOD+HRP | -30.7 | 284 |
| LOD+HRP | 4.95 | 66.43 |
| LOD+HRP+FMN | 36.0 | 264.1 |
| COD-CEH | -8.39 | 51.83 |
| β-CD-FC | 1.19 | 278.1 |
| COD-CEH+HRP | -27.7 | 56.21 |
| β-CD-FC+COD-CEH+HRP | -33.9 | 314.6 |

*Note: GOD: Glucose Oxidase; LOD: Lactate Oxidase; COD: Cholesterol Oxidase; CEH: Cholesterol Esterase; HRP: Horseradish Peroxidase; GA: Glutaraldehyde; FMN: Flavin Mononucleotide; MB: Methylene Blue; Fc: Ferrocene; CS: Chitosan; β-CD: β-Cyclodextrin.*

According to Tab. S1, each layer and the single substance contained in each layer were tested for zeta potential (positivity or negativity, ability for adsorption) and size (degree of cross-linking). All mixed reagents were also formulated and tested in different proportions for control. Zeta potential and particle size were small in single substances and was increased after cross-linking. negative zeta potential in the first and third layers and positive in the middle layer.


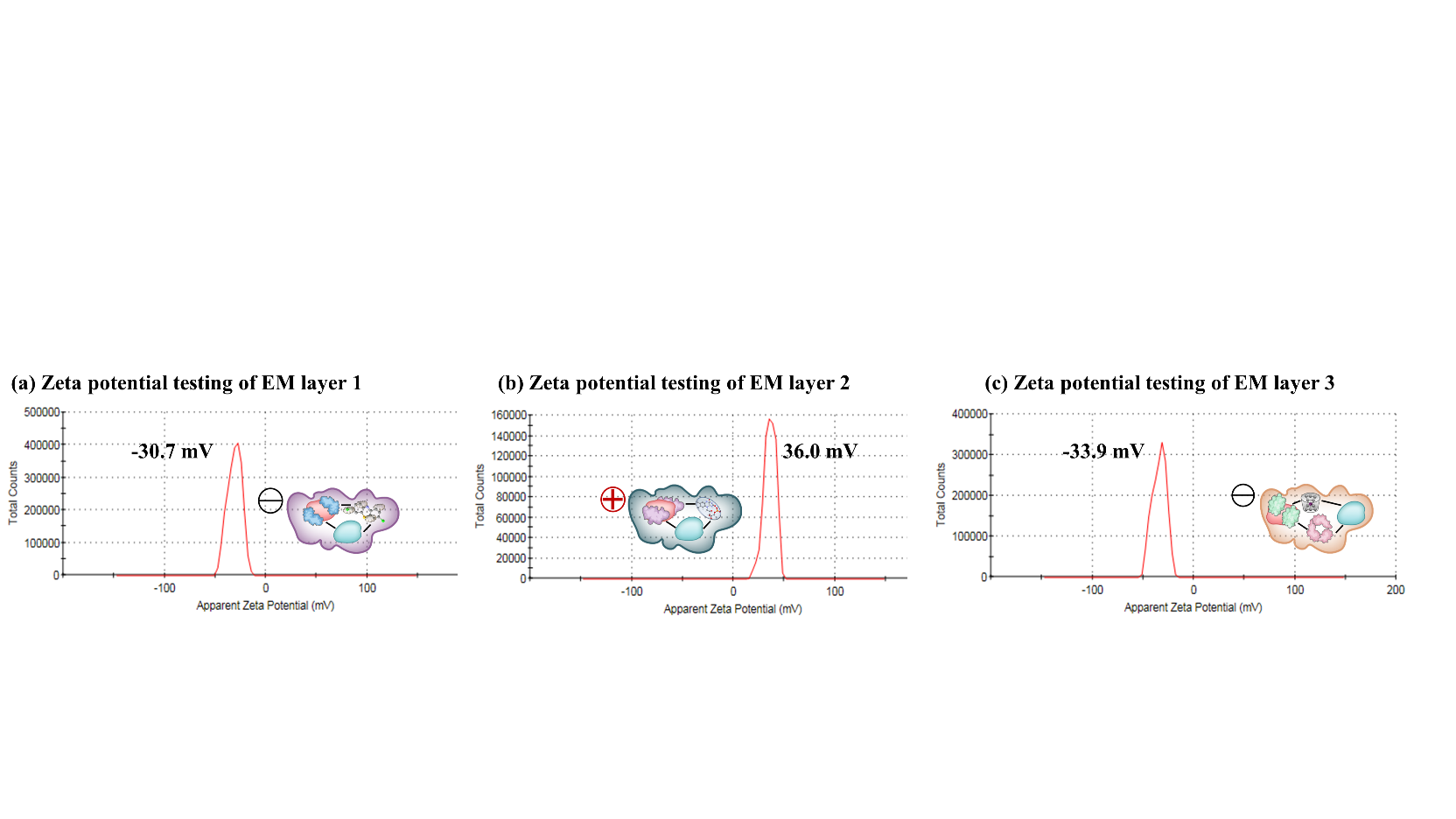


**Fig. S1** (a)-(c) Results of zeta potential testing of EM layer1-3.

Single-layer crosslinking, with more stable material in the EM layer. As shown in Fig. S1, each layer has opposite electrical properties, which was conducive to electrostatic adsorption of layer assembly. The electronegativity and size affect the stability of the test, so the optimal solution ratio and cross-linking conditions are crucial for electrode modification.

EM layer 1 was electronegative and consisted of MB, CS, and GOD for detecting Glu. The sulfur atom in MB exists in an intermediate valence state, has a high affinity for hydroxyl groups, and could be adsorbed by polysaccharides such as CS. The EM layer 2 was electrically positive and consisted of FMN and LOD for detecting Lac. FMN catalyzes redox reactions in biological systems, accelerates reaction processes, and improves reaction rates. The EM layer 3 was electronegative and consisted of Fc, β-CD, CEH, and COD to detect Chol. Fc has important applications in organometallic complexes, functional materials, and biological modifiers owing to its excellent redox activity. This is because of its unique cavity structure (hydrophilic outside and hydrophobic inside). In addition, the EM layers have been cross-linked with each other and assembled by electrostatic charge adsorption, even if diffusion occurs. The detection at the potential corresponding to the paired EM still results in the appearance of characteristic peaks. The reactions occurring at the time of detection are not only within the layers, but in the same sample environment, and simultaneous detection is achieved by separating the peaks of metabolite detection by different EM, so the hydrophilic nature of the molecule does not affect the effective part of current signal detection.

1.3 Analysis on the optimization of modification condition


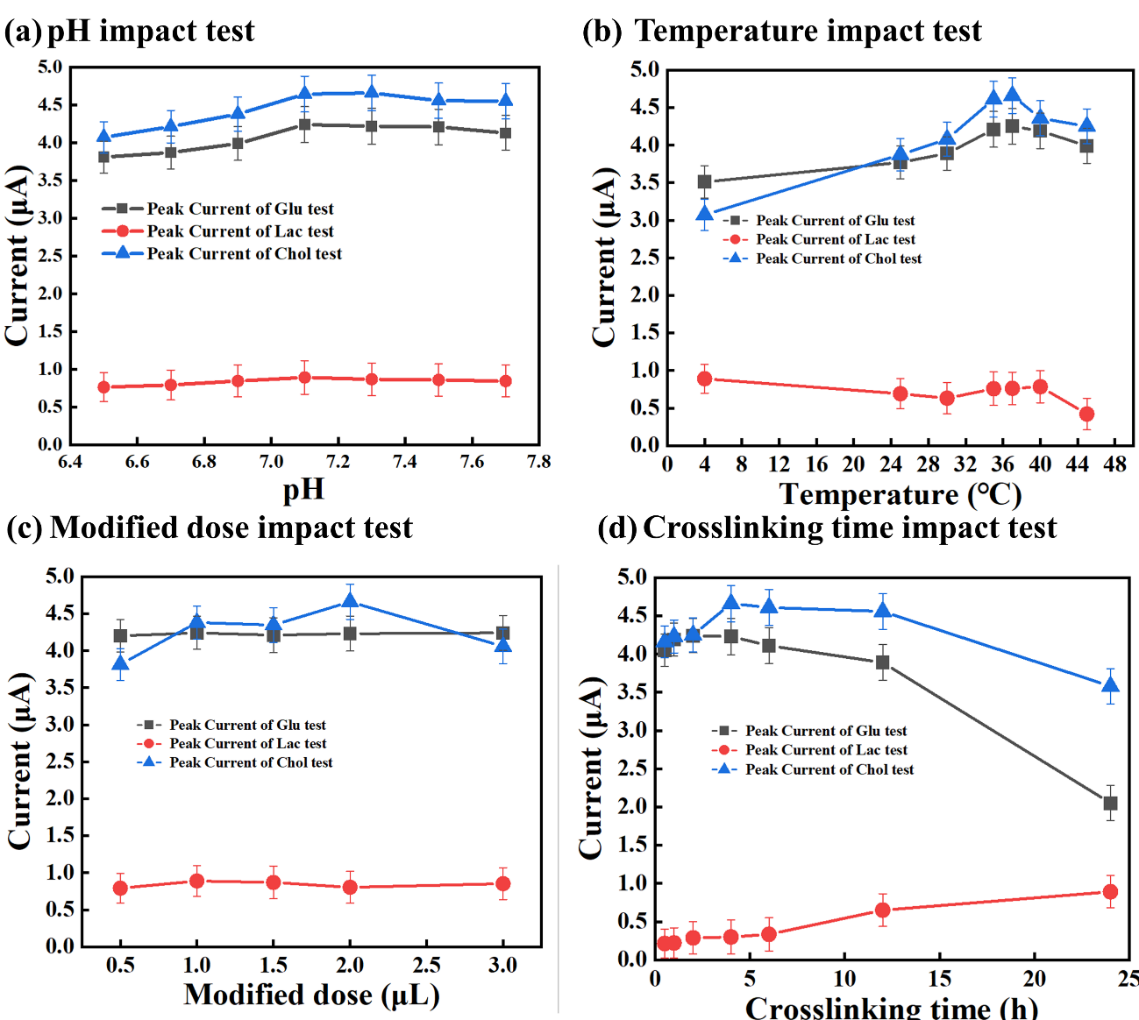


**Fig. S2** (a) – (d) the results of impact test under different factors, these factors included temperature, pH, modified dose, crosslinking time, respectively.

Since the modification system contains a variety of enzymes, the suitable modification conditions for each enzyme were different, so the optimization of temperature, pH, modification method, modification dosage and cross-linking time was crucial. We used a single control factor to explore the influence of the above several influencing factors on the test effect and evaluated the activity of the enzyme in the system by the peak current of the detection curve. The test results were shown in Figure S2. Based on the results of the comprehensive optimization test, we modified 2 μL of each detection solution at 37 °C, pH 7.0, and modified Crosslinking for 4h was the final optimized modification condition.

Repeatability is critical to the accuracy of electrode assay results and is reflected in both electrode fabrication (physical) and electrode testing (biofunctionalization). For electrode fabrication, the repeatability is further reflected in both size and slurry thickness. We use electrodes made using an automated screen printer 3050 (Dongguan LIVO Precision Automation Equipment Co.), so the error in size is very small and negligible compared to the slurry thickness. In addition, the error in paste thickness can be reflected by comparing the DC resistance over the same distance of the electrode.

We have added the experimental results include the impedance repeatability testing (8 mm distance) and metabolite testing under electroenzyme layer modification of the same and different batches of sensors. These results demonstrate excellent sensor repeatability and were shown in Tab. R2-R3 and Fig. R4.

As shown in Tab. R2, the impedance test was carried out randomly among different batches of sensors, and the coefficient of variation of ten times repeatability was less than 3% and the values were close, indicating that the sensors had good consistency.

As shown in Tab. R3, according to the results of different modifications and tests on the same batch of sensors and the same modifications and tests on different batches of sensors, it can be seen that the coefficient of variation of the sensor in eight repeated tests is less than 3.5%, indicating that the sensor also has a good consistency in the test.

**Tab. S2.** Ten resistance test results of two batch sensors (units: kΩ)

| **Batch** | **1** | **2** | **3** | **4** | **5** | **6** | **7** | **8** | **9** | **10** | **Average** | **CV (%)** |
| --- | --- | --- | --- | --- | --- | --- | --- | --- | --- | --- | --- | --- |
| **1** | 0.92 | 0.90 | 0.93 | 0.97 | 0.92 | 0.93 | 0.96 | 0.91 | 0.90 | 0.97 | 0.93 | 2.71 |
| **2** | 0.95 | 0.90 | 0.91 | 0.92 | 0.90 | 0.92 | 0.97 | 0.95 | 0.93 | 0.96 | 0.93 | 2.54 |

**Tab.S3.** The repeatability test results under three layers modification between different batch of EM sensor (units: μA)

| **Batch** | **Sample** | **Current** | **Test 1** | **Test 2** | **Test 3** | **Test 4** | **Test 5** | **Test 6** | **Test 7** | **Test 8** | **Average** | **CV (%)** |
| --- | --- | --- | --- | --- | --- | --- | --- | --- | --- | --- | --- | --- |
| **1** | Glu 5 mM | Peak 1 | 6.23 | 6.32 | 6.34 | 6.57 | 6.21 | 6.09 | 6.14 | 6.01 | 6.24 | 2.79 |
|  |  | Peak 2 | \ | | | | | | | | | |
|  |  | Peak 3 | 4.80 | 4.88 | 4.76 | 4.51 | 5.09 | 4.8 | 4.91 | 4.72 | 4.81 | 3.46 |
|  | Glu 5 mM Lac 1 mM Chol 6 mM | Peak 1 | 6.89 | 6.64 | 6.93 | 7.13 | 6.79 | 6.63 | 6.72 | 6.51 | 6.78 | 2.93 |
|  |  | Peak 2 | 4.00 | 4.09 | 4.2 | 3.76 | 3.98 | 3.94 | 4.1 | 3.98 | 4.01 | 3.27 |
|  |  | Peak 3 | 9.24 | 9.44 | 9.66 | 9.01 | 8.81 | 9.03 | 9.32 | 9.25 | 9.22 | 2.90 |
| **2** | Glu 5 mM Lac 1 mM Chol 6 mM | Peak 1 | 6.80 | 6.78 | 6.89 | 6.61 | 6.73 | 7.15 | 6.89 | 6.53 | 6.80 | 2.80 |
|  |  | Peak 2 | 4.11 | 4.09 | 4.16 | 3.88 | 4.01 | 3.86 | 4.12 | 3.98 | 4.03 | 2.80 |
|  |  | Peak 3 | 9.22 | 9.26 | 9.36 | 9.65 | 8.92 | 9.11 | 9.13 | 9.77 | 9.30 | 3.05 |

Biofunctionalization is reflected in the modified assay. The DPV test curves of the metabolite testing under electroenzyme layer modification of the same and different batches of sensors are shown in Figure R4. It shows the repeatable DPV test results of a single metabolite (5 mM Glu, 1 mM Lac, 4 mM Chol) under different modifications of the same batch of sensors and the DPV test results of different type of metabolite mixed solution under the same modifications of different batches of sensors, The test is stable and has good repeatability. The peak current value is an important basis for converting the concentration, and the excellent repeatability ensures the accuracy of the conversion results.


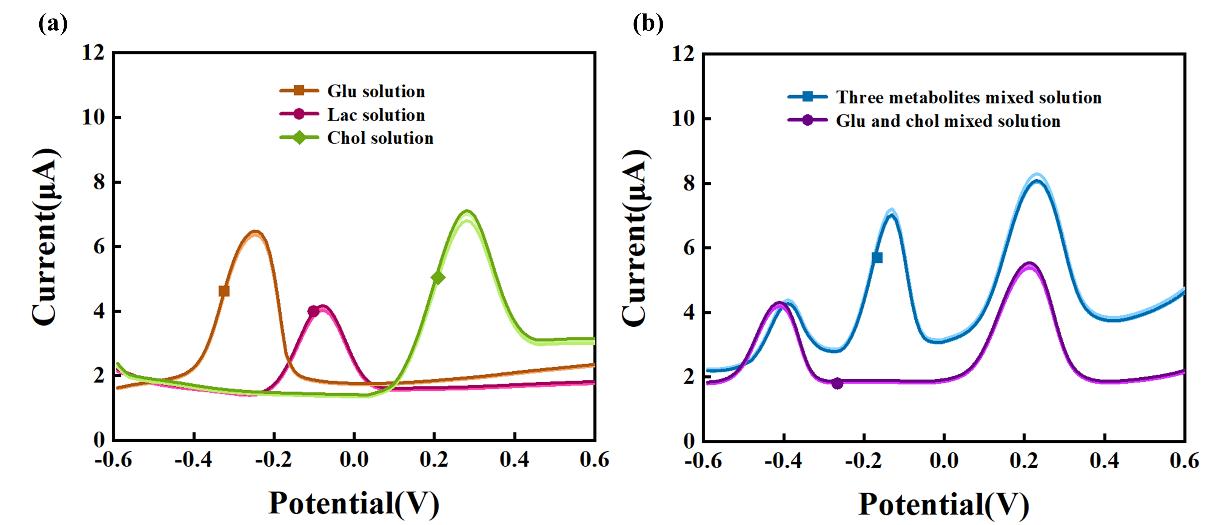


**Fig.S3.** The repeatability test results of EM sensor. (a) Repeatability results of different single samples tested under single layer modification between the same batch; (b) Repeatability results of mixed samples tested under three layers modification between batches

1.4 Gradient test and calibration of EM metabolite sensor in different background

In Fig. 4, (a) glucose gradient test in the background of PBS; Glucose concentrations were 0.01, 0.05, 0.1, 0.25, 0.5, 1, 2.5, 5, 10, 15, 20 and 25 mM. Low lactate concentration (Lac L) was 0.01 mM, high lactate concentration (Lac H) was 10 mM; Low cholesterol concentration (Chol L) was 0.01 mM, high cholesterol concentration (Chol H) was 10 mM; (d) Lactic acid and cholesterol gradient tests for coexistence of multimetabolite; (b) Lactate gradient test with multi-metabolite coexistence under the background of PBS; Lactic acid concentrations were 0.01, 0.05, 0.1, 0.25, 0.5, 1, 2, 4, 6, 8 and 10 mM, respectively. The low concentration of glucose (Glu L) was 0.01 mM, and the high concentration (Glu H) was 25 mM. Chol L and Chol H were 0.01 mM and 10 mM respectively. (e) Glucose and cholesterol gradient tests for coexistence of multimetabolite. (c) Cholesterol gradient test for multi-metabolite coexistence in the context of PBS; Cholesterol concentrations were 0.01, 0.05, 0.1, 0.25, 0.5, 1, 2, 4, 6, 8 and 10 mM, respectively. Glu L was 0.01 mM and Glu H was 25 mM. The Lac L is 0.01 mM and the Lac H is 10 mM. (f) represents a mixed sample with multiple metabolites coexisting and a glucose and lactate gradient test with constant cholesterol concentration.

In Fig. 5, (a) is the glucose gradient test for the coexistence of multiple metabolites in the blood background; Glucose concentrations were 5, 10, 15, 20 and 25 mM. The Lac L is 1 mM and the Lac H is 10 mM. Chol L and Chol H are 1 mM and 10 mM respectively. (d) Lactic acid and cholesterol gradient tests for coexistence of multimetabolite; (b) A glucose gradient test for the coexistence of multiple metabolites in the blood context; Lactic acid concentrations were 1, 2, 4, 6 and 10 mM, respectively. Glu L is 5 mM and Glu H is 25 mM. Chol L and Chol H are 1 mM and 10 mM respectively. (e) Multi-metabolite coexistence of glucose and cholesterol gradient tests. (c) A cholesterol gradient test for the coexistence of multiple metabolites in the blood context; Cholesterol concentrations were 1, 2, 4, 6 and 10 mM, respectively. Glu L is 5 mM and Glu H is 25 mM. The Lac L is 1 mM and the Lac H is 10 mM. (f) Glucose and lactate gradient tests for coexistence of multimetabolite.

To explore this interference, we performed gradient experiments with glucose, lactic acid and cholesterol tests while controlling for other variables. According to the test results, we found that the concentration gradient of metabolites is linear with the current intensity, but the change law is slightly different. To quantify this difference, further analysis of the detection data is required.

When the samples were mixed with Glu, Lac, and Chol, the combined gradient test results at high or low concentrations of the different substances were used to analyze the mutual interference between the tested substances. The DPV results for mixed modifications of the three metabolites coexisting in PBS were shown in Fig. 3 (A). In addition, plasma contains more proteins, bioelectrolytes and other components. The gradient test based on plasma was more disturbed by intracellular Glu metabolism than was the gradient test based on PBS. To better apply the sensor under clinical conditions, we conducted a gradient experiment with an accuracy of 0.01 mm in PBS background and tested five concentrations under a plasma background for calibration. The test results were shown in Fig. 3 (B).

For the calibration of PBS and plasma, the matrix [P1] of the partial gradient test under PBS, matrix [P2] of the full gradient (11 groups of concentrations) test, and matrix [P1'] of the partial gradient (5 groups of concentrations) test under plasma were obtained. The full gradient matrix [P2`], corrected by the coefficient matrix, was used to correct the test results for Glu and Chol in the plasma. The detail calculation processes is mainly divided into the following steps: The gradient test of 11 groups of concentrations of 0.01-25 mM (Glu), 0.01-10 mM (Lac) and 0.01-10 mM (Chol) are carried out under the background of PBS, the concentration of two metabolites is controlled unchanged (at a low or high level), and the gradient increases the concentration of one metabolite; Controlling the concentration of one metabolite unchanged (at a low or high level), the gradient increases the concentration of the other two metabolites. The detection data of three peak currents under 11 concentration tests (observation value of 1884 groups) in PBS background can be obtained (Figure 4), and the linear regression matrix can be calculated through multiple linear regression. Due to the complex background of blood, gradient tests were performed against the plasma background for 5 sets of concentrations (observation value of 150 groups) of 5-25 mM (Glu), 2-10 mM (Lac) and 2-10 mM (Chol), which were included in the 11 sets of concentrations in the PBS background test. The detection data of three peak currents under 5 sets of plasma background concentration tests can be obtained (Figure 5). The linear regression matrix [P1']and the linear regression matrix [P1] under the PBS background of the same 5 groups of concentrations can be calculated through multiple linear regression. [P1] can be transformed into [P1']by multiplying the coefficient matrix [V], and likewise [P2] can be transformed into [P2'] by multiplying the coefficient matrix [V], and [P2] can be thought of as a matrix equation for 11 sets of concentration tests in the plasma background, whose concentrations are 0.01-25 mM (Glu), 0.01-10 mM (Lac) and 0.01-10 mM (Chol), respectively.

The coefficient values from the Glu analysis were reported in the first row of the coefficient matrix according to the order of i1, i2, i3 and the intercept; same analyses were reported for Lac and Chol; and the single vector was reported in the fourth row to form the coefficient matrix. Three sets of coefficient matrices, [P1], [P1`] and [P2], were obtained by linear regression analysis. Through the relationship between [P1] and [P1'], the value of transformation matrix [V] was obtained according to the product of [P1'] and [P1]-1, and the value of full gradient coefficient matrix [P2'] under the background of blood plasma was calculated according to the product of [P2'] and [P2]^-1^.

The calculation processes were shown in Fig. 3(c) and Eqs. S1 -S8.

$\left[ Glu \right]= \left[ \begin{matrix} \begin{matrix} g_{1} & g_{2} \end{matrix} & g_{3} & g_{intercept} \end{matrix} \right]*\left[ \begin{matrix} \begin{matrix} i_{1} \\ i_{2} \end{matrix} \\ i_{3} \\ 1 \end{matrix} \right]$ Eq. S1

$\left[ Lac \right]=\left[ \begin{matrix} \begin{matrix} l_{1} & l_{2} \end{matrix} & l_{3} & l_{intercept} \end{matrix} \right]*\left[ \begin{matrix} \begin{matrix} i_{1} \\ i_{2} \end{matrix} \\ i_{3} \\ 1 \end{matrix} \right]$ Eq. S2

$\left[ Chol \right]= \left[ \begin{matrix} \begin{matrix} c_{1} & c_{2} \end{matrix} & c_{3} & c_{intercept} \end{matrix} \right]*\left[ \begin{matrix} \begin{matrix} i_{1} \\ i_{2} \end{matrix} \\ i_{3} \\ 1 \end{matrix} \right]$ Eq. S3

$\left[ P \right]=\left[ \begin{matrix} \begin{matrix} g_{1} & g_{2} \\ l_{1} & l_{1} \end{matrix} & \begin{matrix} g_{3} & g_{intercept} \\ l_{3} & l_{intercept} \end{matrix} \\ \begin{matrix} c_{1} & c_{2} \\ 0 & 0 \end{matrix} & \begin{matrix} c_{3} & c_{intercept} \\ 0 & 1 \end{matrix} \end{matrix} \right]$ Eq. S4

$\left[ Glu` \right]= \left[ \begin{matrix} \begin{matrix} {g`}_{1} & {g`}_{2} \end{matrix} & {g`}_{3} & {g`}_{intercept} \end{matrix} \right]*\left[ \begin{matrix} \begin{matrix} {i`}_{1} \\ {i`}_{2} \end{matrix} \\ {i`}_{3} \\ 1 \end{matrix} \right]$ Eq. S5

$\left[ Lac` \right]=\left[ \begin{matrix} \begin{matrix} l_{`1} & {l`}_{2} \end{matrix} & {l`}_{3} & {l`}_{intercept} \end{matrix} \right]*\left[ \begin{matrix} \begin{matrix} {i`}_{1} \\ {i`}_{2} \end{matrix} \\ {i`}_{3} \\ 1 \end{matrix} \right]$ Eq. S6

$\left[ Chol` \right]= \left[ \begin{matrix} \begin{matrix} {c`}_{1} & c_{`2} \end{matrix} & {c`}_{3} & {c`}_{intercept} \end{matrix} \right]*\left[ \begin{matrix} \begin{matrix} {i`}_{1} \\ {i`}_{2} \end{matrix} \\ {i`}_{3} \\ 1 \end{matrix} \right]$ Eq. S7

$\left[ P^{`} \right]=\left[ \begin{matrix} \begin{matrix} {g`}_{1} & {g`}_{2} \\ {l`}_{1} & {l`}_{1} \end{matrix} & \begin{matrix} {g`}_{3} & {g`}_{intercept} \\ {l`}_{3} & {l`}_{intercept} \end{matrix} \\ \begin{matrix} {c`}_{1} & {c`}_{2} \\ 0 & 0 \end{matrix} & \begin{matrix} {c`}_{3} & {c`}_{intercept} \\ 0 & 1 \end{matrix} \end{matrix} \right]$ Eq. S8

The results of linear regression analysis of the three groups of data were shown in Tab. S2. Similarly, the fitting analysis results of the partial gradient in the plasma background and the full gradient in the PBS background were shown in Tab. S2 and Tab. S3. The analysis results of the partial gradient test in the plasma background were consistent with the PBS experimental results, but the values were different. By multiplying the regression coefficient [P1] by the correction coefficient matrix V, we obtained the full gradient regression coefficient matrix under the plasma background. By comparing the fitting results of the partial gradient and full gradient of PBS, the fitting correlation of Glu and Chol was improved. Therefore, the full gradient matrix [P2`], corrected by the coefficient matrix, was used to correct the test results of Glu and Chol in the plasma background. The calculation process was shown in Eqs. S9-S19. The concentration of metabolites in the sample could be calculated by substituting the peak value into Eq. S20.

$\left[ \begin{matrix} \mathrm{Glu} \\ \mathrm{Lac} \\ \begin{matrix} \mathrm{Chol} \\ \vec{n} \end{matrix} \end{matrix} \right]= \left[ P \right]*\left[ \begin{matrix} \begin{matrix} i_{1} \\ i_{2} \end{matrix} \\ i_{3} \\ 1 \end{matrix} \right]\underset{\to}{In PBS \underset{\to}{*\left[ V \right]} In Plasma}\left[ \begin{matrix} Glu` \\ Lac` \\ \begin{matrix} Chol` \\ \vec{n} \end{matrix} \end{matrix} \right]= \left[ P` \right]*\left[ \begin{matrix} \begin{matrix} {i`}_{1} \\ {i`}_{2} \end{matrix} \\ {i`}_{3} \\ 1 \end{matrix} \right]$ Eq. S11

$\left[ P_{1} \right]*\left[ V \right]=\left[ P_{1}` \right]$ Eq. S12

$\left[ V \right]=\left[ {P_{1}}^{-1} \right]*\left[ P_{1}` \right]$ Eq. S13

$\left[ P_{2}` \right]=\left[ P_{2} \right]*\left[ V \right]$ Eq. S14

$\left[ P_{1} \right]=\left[ \begin{matrix} \begin{matrix} 4.202 & -0.132 \\ 0.039 & 2.012 \end{matrix} & \begin{matrix} -0.263 & -20.502 \\ -0.102 & -8.691 \end{matrix} \\ \begin{matrix} -0.010 & -0.092 \\ 0 & 0 \end{matrix} & \begin{matrix} 1.673 & -9.017 \\ 0 & 1 \end{matrix} \end{matrix} \right]$ Eq. S15

$\left[ {P_{1}}^{`} \right]=\left[ \begin{matrix} \begin{matrix} 4.240 & 0.018 \\ 0.006 & 2.205 \end{matrix} & \begin{matrix} -0.004 & -22.417 \\ 0.023 & -6.474 \end{matrix} \\ \begin{matrix} -0.106 & -0.036 \\ 0 & 0 \end{matrix} & \begin{matrix} 2.081 & -12.450 \\ 0 & 1 \end{matrix} \end{matrix} \right]$ Eq. S16)

$\left[ V \right]=\left[ \begin{matrix} \begin{matrix} 1.005 & -0.041 \\ -0.014 & 1.093 \end{matrix} & \begin{matrix} 0.075 & -0.542 \\ -0.053 & 1.214 \end{matrix} \\ \begin{matrix} -0.058 & 0.039 \\ 0 & 0 \end{matrix} & \begin{matrix} 1.242 & -1.989 \\ 0 & 1 \end{matrix} \end{matrix} \right]$ Eq. S17

$\left[ P_{2} \right]=\left[ \begin{matrix} \begin{matrix} 3.663 & -0.193 \\ 0.005 & 1.786 \end{matrix} & \begin{matrix} -0.155 & -15.864 \\ -0.086 & -6.575 \end{matrix} \\ \begin{matrix} -0.001 & -0.138 \\ 0 & 0 \end{matrix} & \begin{matrix} 1.650 & -8.676 \\ 0 & 1 \end{matrix} \end{matrix} \right]$ Eq. S18

$\left[ {P_{2}}^{`} \right]=\left[ \begin{matrix} \begin{matrix} 3.620 & -0.3 \\ 0.028 & 1.632 \end{matrix} & \begin{matrix} -0.357 & -14.246 \\ -0.001 & -8.544 \end{matrix} \\ \begin{matrix} 0.072 & -0.176 \\ 0 & 0 \end{matrix} & \begin{matrix} 1.317 & -5.805 \\ 0 & 1 \end{matrix} \end{matrix} \right]$ Eq. S19

$\left[ \begin{matrix} Glu` \\ Lac` \\ \begin{matrix} Chol` \\ \vec{n} \end{matrix} \end{matrix} \right]= \left[ \begin{matrix} \begin{matrix} 3.620 & -0.3 \\ 0.028 & 1.632 \end{matrix} & \begin{matrix} -0.357 & -14.246 \\ -0.001 & -8.544 \end{matrix} \\ \begin{matrix} 0.072 & -0.176 \\ 0 & 0 \end{matrix} & \begin{matrix} 1.317 & -5.805 \\ 0 & 1 \end{matrix} \end{matrix} \right]*\left[ \begin{matrix} \begin{matrix} {i`}_{1} \\ {i`}_{2} \end{matrix} \\ {i`}_{3} \\ 1 \end{matrix} \right]$ Eq. S20

The matrix calibration results and analysis in the background of PBS and plasma were shown in the Tab. S4 and Tab. S5. In each gradient test, the three metabolite concentrations had different weight relationships with the three peak currents. In the partial gradient test in the PBS background, the correlation coefficient between Glu and the peak current value i_1_ at a potential of -0.42 ~ -0.41 V was 4.202, which was the largest; the standard error was 0.013, and the fitting correlation coefficient was 0.999. The correlation coefficient between Lac and the peak current value i_2_ at a potential of -0.19 ~ -0.18 V was 2.012, which was the largest; the standard error was 0.035, and the fitting correlation coefficient was 0.979. The correlation coefficient between Chol and the peak current value i_3_ at a potential of 0.2~0.21 V was 1.673, which was the largest; the standard error was 0.019, and the fitting correlation coefficient was 0.990.

**Tab. S4** Fitting analysis of standard gradient tests for linear regression model in PBS and plasma

| Matrix | Metabolite | Variable | Coefficient | Standard Error | t Stat | P value | Lower 95% | Upper 95% | Fitting |
| --- | --- | --- | --- | --- | --- | --- | --- | --- | --- |
| P_1_ | Glu | Intercept | -20.502 | 0.172 | -118.947 | 0.000 | -20.842 | -20.161 | 0.999 |
|  |  | I 1 | 4.202 | 0.013 | 320.344 | 0.000 | 4.176 | 4.228 |  |
|  |  | I 2 | -0.132 | 0.014 | -9.358 | 0.000 | -0.160 | -0.104 |  |
|  |  | I 3 | -0.263 | 0.011 | -24.020 | 0.000 | -0.285 | -0.242 |  |
|  | Lac | Intercept | -8.691 | 0.420 | -20.681 | 0.000 | -9.521 | -7.860 | 0.979 |
|  |  | I 1 | 0.039 | 0.032 | 1.219 | 0.225 | -0.024 | 0.102 |  |
|  |  | I 2 | 2.012 | 0.035 | 58.297 | 0.000 | 1.943 | 2.080 |  |
|  |  | I 3 | 0.102 | 0.027 | 3.822 | 0.000 | 0.049 | 0.155 |  |
|  | Chol | Intercept | -9.017 | 0.298 | -30.251 | 0.000 | -9.606 | -8.428 | 0.990 |
|  |  | I 1 | -0.010 | 0.023 | -0.456 | 0.649 | -0.055 | 0.034 |  |
|  |  | I 2 | -0.092 | 0.024 | -3.766 | 0.000 | -0.141 | -0.044 |  |
|  |  | I 3 | 1.673 | 0.019 | 88.215 | 0.000 | 1.635 | 1.710 |  |
| P_1_` | Glu | Intercept | -22.417 | 0.210 | -106.540 | 0.000 | -22.832 | -22.001 | 0.999 |
|  |  | I 1 | 4.240 | 0.014 | 303.648 | 0.000 | 4.212 | 4.267 |  |
|  |  | I 2 | -0.018 | 0.016 | -1.136 | 0.258 | -0.049 | 0.013 |  |
|  |  | I 3 | -0.004 | 0.017 | -0.234 | 0.815 | -0.038 | 0.030 |  |
|  | Lac | Intercept | -6.474 | 0.694 | -9.331 | 0.000 | -7.845 | -5.103 | 0.961 |
|  |  | I 1 | 0.006 | 0.046 | 0.124 | 0.901 | -0.085 | 0.097 |  |
|  |  | I 2 | 2.205 | 0.052 | 42.531 | 0.000 | 2.102 | 2.307 |  |
|  |  | I 3 | 0.023 | 0.057 | 0.405 | 0.686 | -0.089 | 0.136 |  |
|  | Chol | Intercept | -12.450 | 0.308 | -40.483 | 0.000 | -13.058 | -11.842 | 0.989 |
|  |  | I 1 | -0.105 | 0.020 | -5.167 | 0.000 | -0.146 | -0.065 |  |
|  |  | I 2 | -0.036 | 0.023 | -1.579 | 0.116 | -0.082 | 0.009 |  |
|  |  | I 3 | 2.081 | 0.025 | 82.448 | 0.000 | 2.031 | 2.131 |  |
| P_2_ | Glu | Intercept | -15.863 | 0.141 | -112.166 | 0.000 | -16.140 | -15.586 | 0.990 |
|  |  | I 1 | 3.662 | 0.012 | 304.939 | 0.000 | 3.639 | 3.686 |  |
|  |  | I 2 | -0.193 | 0.015 | -13.176 | 0.000 | -0.221 | -0.164 |  |
|  |  | I 3 | -0.155 | 0.013 | -11.566 | 0.000 | -0.181 | -0.129 |  |
|  | Lac | Intercept | -6.575 | 0.079 | -83.650 | 0.000 | -6.729 | -6.421 | 0.981 |
|  |  | I 1 | -0.005 | 0.007 | -0.811 | 0.418 | -0.019 | 0.008 |  |
|  |  | I 2 | 1.786 | 0.008 | 219.814 | 0.000 | 1.770 | 1.802 |  |
|  |  | I 3 | 0.086 | 0.007 | 11.540 | 0.000 | 0.071 | 0.101 |  |
|  | Chol | Intercept | -8.676 | 0.068 | -126.941 | 0.000 | -8.810 | -8.542 | 0.985 |
|  |  | I 1 | -0.001 | 0.006 | -0.196 | 0.845 | -0.013 | 0.010 |  |
|  |  | I 2 | -0.138 | 0.007 | -19.539 | 0.000 | -0.152 | -0.124 |  |
|  |  | I 3 | 1.650 | 0.006 | 254.866 | 0.000 | 1.637 | 1.663 |  |

Tab. S5 Residual analysis of standard gradient tests for linear regression model in PBS and plasma

| Matrix | Metabolites | Analysis | df | SS | MS | | F | Significance F |  |
| --- | --- | --- | --- | --- | --- | --- | --- | --- | --- |
| P_1_ | Glu | Regression | 3.000 | 7464.220 | 2488.073 | | 34225.230 | 0.000 |  |
|  |  | Residual | 146.000 | 10.614 | 0.073 | |  |  |  |
|  |  | Total | 149.000 | 7474.833 |  | |  |  |  |
|  | Lac | Regression | 3.000 | 1472.908 | 490.969 | | 1136.135 | 0.000 |  |
|  |  | Residual | 146.000 | 63.092 | 0.432 | |  |  |  |
|  |  | Total | 149.000 | 1536.000 |  | |  |  |  |
|  | Chol | Regression | 3.000 | 1709.248 | 569.749 | | 2620.364 | 0.000 |  |
|  |  | Residual | 146.000 | 31.745 | 0.217 | |  |  |  |
|  |  | Total | 149.000 | 1740.993 |  | |  |  |  |
| P_1_` | Glu | Regression | 3.000 | 7488.190 | 2496.063 | | 30856.229 | 0.000 |  |
|  |  | Residual | 146.000 | 11.810 | 0.081 | |  |  |  |
|  |  | Total | 149.000 | 7500.000 |  | |  |  |  |
|  | Lac | Regression | 3.000 | 1592.412 | 530.804 | | 603.462 | 0.000 |  |
|  |  | Residual | 146.000 | 128.421 | 0.880 | |  |  |  |
|  |  | Total | 149.000 | 1720.833 |  | |  |  |  |
|  | Chol | Regression | 3.000 | 1174.768 | 391.589 | | 2265.873 | 0.000 |  |
|  |  | Residual | 146.000 | 25.232 | 0.173 | |  |  |  |
|  |  | Total | 149.000 | 1200.000 |  | |  |  |  |
| P_2_ | Glu | Regression | 3.000 | 127115.273 | 42371.758 | | 30996.325 | 0.000 |  |
|  |  | Residual | 1880.000 | 2569.947 | 1.367 | |  |  |  |
|  |  | Total | 1883.000 | 129685.220 |  | |  |  |  |
|  | Lac | Regression | 3.000 | 20563.510 | 6854.503 | | 16232.599 | 0.000 |  |
|  |  | Residual | 1880.000 | 793.863 | 0.422 | |  |  |  |
|  |  | Total | 1883.000 | 21357.374 |  | |  |  |  |
|  | Chol | Regression | 3.000 | 20823.043 | 6941.014 | | 21739.377 | 0.000 |  |
|  |  | Residual | 1880.000 | 600.252 | 0.319 | |  |  |  |
|  |  | Total | 1883.000 | 21423.295 |  | |  |  |  |
| * p<0.05 ** p<0.01 | | | | | |  | | | |

1.5 Clinical validation data of EM metabolite sensor

The test data of 242 patients were shown in Tab. S6.

Tab. S6 Clinical validation data of EM metabolite sensor

| serial number | Glu-Concentration | Glu-Current i1 | Error Bar | Lac-Concentration | Lac-Current i2 | Error Bar | Chol-Concentration | Chol-Current i3 | Error Bar |
| --- | --- | --- | --- | --- | --- | --- | --- | --- | --- |
| 1 | 4.93 | 6.603 | 0.059 | 0.56 | 5.494 | 0.011 | 5.10 | 8.651 | 0.074 |
| 2 | 4.54 | 6.366 | 0.062 | 0.56 | 5.429 | 0.012 | 3.02 | 7.119 | 0.033 |
| 3 | 4.78 | 6.451 | 0.061 | 0.57 | 5.437 | 0.011 | 3.66 | 7.561 | 0.030 |
| 4 | 4.66 | 6.508 | 0.064 | 0.57 | 5.357 | 0.011 | 4.41 | 8.175 | 0.038 |
| 5 | 4.14 | 6.271 | 0.064 | 0.57 | 5.347 | 0.012 | 3.46 | 7.433 | 0.028 |
| 6 | 4.62 | 6.481 | 0.060 | 0.58 | 5.536 | 0.011 | 4.12 | 7.964 | 0.045 |
| 7 | 4.54 | 6.542 | 0.058 | 0.59 | 5.514 | 0.010 | 5.71 | 9.118 | 0.069 |
| 8 | 4.78 | 6.603 | 0.062 | 0.59 | 5.481 | 0.012 | 5.65 | 9.070 | 0.060 |
| 9 | 5.00 | 6.508 | 0.070 | 0.59 | 5.514 | 0.013 | 3.15 | 7.218 | 0.029 |
| 10 | 5.11 | 6.671 | 0.064 | 0.60 | 5.490 | 0.012 | 5.45 | 8.902 | 0.076 |
| 11 | 5.51 | 6.676 | 0.064 | 0.61 | 5.514 | 0.011 | 3.66 | 7.582 | 0.032 |
| 12 | 5.75 | 6.863 | 0.061 | 0.61 | 5.514 | 0.005 | 5.26 | 8.790 | 0.043 |
| 13 | 5.34 | 6.767 | 0.061 | 0.62 | 5.432 | 0.017 | 5.36 | 8.884 | 0.062 |
| 14 | 5.65 | 6.857 | 0.063 | 0.63 | 5.514 | 0.011 | 5.97 | 9.286 | 0.055 |
| 15 | 6.20 | 6.880 | 0.063 | 0.64 | 5.527 | 0.009 | 4.11 | 7.886 | 0.037 |
| 16 | 5.10 | 6.722 | 0.060 | 0.64 | 5.540 | 0.012 | 5.75 | 9.172 | 0.049 |
| 17 | 5.63 | 6.782 | 0.062 | 0.52 | 5.426 | 0.017 | 4.90 | 8.492 | 0.037 |
| 18 | 4.96 | 6.523 | 0.058 | 0.53 | 5.449 | 0.012 | 3.79 | 7.673 | 0.045 |
| 19 | 4.90 | 6.511 | 0.058 | 0.53 | 5.449 | 0.012 | 3.89 | 7.745 | 0.033 |
| 20 | 5.93 | 6.913 | 0.065 | 0.53 | 5.449 | 0.005 | 4.84 | 8.513 | 0.085 |
| 21 | 4.82 | 6.462 | 0.059 | 0.55 | 5.436 | 0.013 | 3.31 | 7.330 | 0.031 |
| 22 | 4.38 | 6.401 | 0.062 | 0.55 | 5.463 | 0.011 | 4.36 | 8.106 | 0.040 |
| 23 | 5.15 | 6.665 | 0.064 | 0.56 | 5.451 | 0.011 | 4.98 | 8.572 | 0.035 |
| 24 | 5.00 | 6.660 | 0.061 | 0.56 | 5.464 | 0.012 | 5.52 | 8.977 | 0.083 |
| 25 | 6.83 | 7.016 | 0.058 | 0.56 | 5.462 | 0.011 | 3.54 | 7.454 | 0.033 |
| 26 | 4.70 | 6.520 | 0.060 | 0.56 | 5.447 | 0.010 | 4.71 | 8.372 | 0.029 |
| 27 | 6.31 | 6.956 | 0.061 | 0.56 | 5.471 | 0.003 | 4.44 | 8.162 | 0.029 |
| 28 | 4.22 | 6.399 | 0.058 | 0.56 | 5.466 | 0.011 | 4.76 | 8.425 | 0.032 |
| 29 | 5.80 | 6.676 | 0.062 | 0.56 | 5.461 | 0.005 | 2.92 | 6.986 | 0.032 |
| 30 | 5.74 | 6.922 | 0.063 | 0.57 | 5.470 | 0.011 | 5.96 | 9.333 | 0.128 |
| 31 | 5.49 | 6.797 | 0.101 | 0.57 | 5.470 | 0.011 | 5.27 | 8.808 | 0.049 |
| 32 | 5.95 | 6.727 | 0.087 | 0.57 | 5.481 | 0.011 | 2.76 | 6.892 | 0.031 |
| 33 | 4.54 | 6.460 | 0.064 | 0.58 | 5.480 | 0.010 | 4.37 | 8.130 | 0.036 |
| 34 | 4.47 | 6.325 | 0.058 | 0.59 | 5.484 | 0.012 | 2.84 | 6.972 | 0.098 |
| 35 | 4.86 | 6.516 | 0.058 | 0.59 | 5.480 | 0.012 | 4.09 | 7.899 | 0.034 |
| 36 | 4.74 | 6.572 | 0.063 | 0.59 | 5.480 | 0.019 | 5.11 | 8.689 | 0.069 |
| 37 | 5.69 | 6.674 | 0.059 | 0.60 | 5.493 | 0.011 | 3.30 | 7.275 | 0.040 |
| 38 | 5.49 | 6.718 | 0.108 | 0.61 | 5.486 | 0.012 | 4.37 | 8.112 | 0.030 |
| 39 | 6.85 | 6.973 | 0.059 | 0.61 | 5.496 | 0.012 | 2.91 | 6.974 | 0.032 |
| 40 | 5.05 | 6.609 | 0.059 | 0.62 | 5.505 | 0.012 | 4.55 | 8.253 | 0.037 |
| 41 | 7.98 | 7.334 | 0.060 | 0.63 | 5.511 | 0.011 | 3.26 | 7.255 | 0.033 |
| 42 | 5.00 | 6.600 | 0.062 | 0.64 | 5.515 | 0.012 | 4.84 | 8.449 | 0.037 |
| 43 | 4.88 | 6.712 | 0.058 | 0.64 | 5.518 | 0.011 | 6.48 | 9.721 | 0.069 |
| 44 | 5.15 | 6.580 | 0.060 | 0.64 | 5.516 | 0.005 | 3.73 | 7.638 | 0.040 |
| 45 | 4.70 | 6.439 | 0.061 | 0.65 | 5.520 | 0.006 | 3.25 | 7.307 | 0.026 |
| 46 | 7.15 | 6.964 | 0.062 | 0.65 | 5.520 | 0.011 | 2.08 | 6.303 | 0.030 |
| 47 | 7.14 | 7.171 | 0.059 | 0.65 | 5.520 | 0.012 | 4.61 | 8.244 | 0.055 |
| 48 | 6.38 | 6.961 | 0.059 | 0.65 | 5.512 | 0.011 | 4.22 | 7.998 | 0.035 |
| 49 | 5.80 | 6.811 | 0.062 | 0.67 | 5.529 | 0.019 | 4.49 | 8.197 | 0.041 |
| 50 | 7.91 | 7.316 | 0.066 | 0.67 | 5.531 | 0.019 | 2.99 | 7.080 | 0.035 |
| 51 | 4.83 | 6.697 | 0.059 | 0.67 | 5.540 | 0.020 | 6.15 | 9.505 | 0.066 |
| 52 | 8.16 | 7.409 | 0.061 | 0.67 | 5.533 | 0.018 | 3.89 | 7.700 | 0.044 |
| 53 | 3.30 | 6.059 | 0.061 | 0.68 | 5.526 | 0.019 | 3.14 | 7.261 | 0.028 |
| 54 | 4.69 | 6.494 | 0.062 | 0.68 | 5.549 | 0.018 | 4.45 | 8.171 | 0.039 |
| 55 | 5.32 | 6.731 | 0.058 | 0.69 | 5.536 | 0.020 | 5.37 | 8.850 | 0.059 |
| 56 | 5.57 | 6.776 | 0.064 | 0.70 | 5.594 | 0.019 | 4.65 | 8.342 | 0.028 |
| 57 | 5.27 | 6.740 | 0.062 | 0.70 | 5.593 | 0.018 | 5.69 | 9.092 | 0.223 |
| 58 | 4.18 | 6.256 | 0.063 | 0.70 | 5.591 | 0.026 | 2.68 | 6.886 | 0.035 |
| 59 | 4.89 | 6.616 | 0.059 | 0.71 | 5.594 | 0.020 | 5.17 | 8.729 | 0.041 |
| 60 | 5.35 | 6.753 | 0.062 | 0.72 | 5.605 | 0.029 | 5.16 | 8.730 | 0.045 |
| 61 | 5.33 | 6.721 | 0.063 | 0.73 | 5.608 | 0.004 | 4.88 | 8.512 | 0.103 |
| 62 | 4.86 | 6.470 | 0.062 | 0.73 | 5.599 | 0.018 | 3.46 | 7.424 | 0.031 |
| 63 | 6.45 | 7.142 | 0.063 | 0.73 | 5.612 | 0.026 | 6.51 | 9.716 | 0.061 |
| 64 | 5.47 | 6.698 | 0.062 | 0.73 | 5.584 | 0.019 | 3.70 | 7.654 | 0.047 |
| 65 | 5.43 | 6.765 | 0.063 | 0.73 | 5.614 | 0.018 | 5.13 | 8.697 | 0.067 |
| 66 | 6.69 | 7.073 | 0.058 | 0.74 | 5.607 | 0.009 | 4.83 | 8.430 | 0.033 |
| 67 | 5.49 | 6.747 | 0.059 | 0.74 | 5.605 | 0.007 | 4.63 | 8.322 | 0.034 |
| 68 | 5.53 | 6.713 | 0.057 | 0.75 | 5.622 | 0.018 | 4.39 | 8.103 | 0.027 |
| 69 | 6.77 | 7.100 | 0.060 | 0.75 | 5.622 | 0.019 | 4.74 | 8.376 | 0.031 |
| 70 | 5.68 | 6.690 | 0.061 | 0.76 | 5.552 | 0.020 | 3.69 | 7.556 | 0.034 |
| 71 | 5.10 | 6.563 | 0.061 | 0.76 | 5.557 | 0.020 | 3.58 | 7.536 | 0.030 |
| 72 | 5.73 | 6.773 | 0.059 | 0.77 | 5.561 | 0.038 | 4.53 | 8.198 | 0.031 |
| 73 | 5.92 | 6.920 | 0.067 | 0.77 | 5.553 | 0.018 | 5.28 | 8.815 | 0.051 |
| 74 | 4.57 | 6.514 | 0.063 | 0.77 | 5.556 | 0.020 | 5.24 | 8.761 | 0.057 |
| 75 | 6.21 | 6.951 | 0.060 | 0.78 | 5.532 | 0.018 | 4.84 | 8.456 | 0.046 |
| 76 | 5.44 | 6.624 | 0.077 | 0.78 | 5.569 | 0.021 | 3.52 | 7.448 | 0.033 |
| 77 | 5.56 | 6.640 | 0.066 | 0.78 | 5.563 | 0.020 | 3.11 | 7.154 | 0.038 |
| 78 | 8.36 | 7.446 | 0.058 | 0.79 | 5.572 | 0.018 | 3.57 | 7.464 | 0.040 |
| 79 | 6.34 | 6.852 | 0.074 | 0.79 | 5.575 | 0.018 | 3.09 | 7.127 | 0.052 |
| 80 | 6.47 | 7.028 | 0.062 | 0.80 | 5.576 | 0.020 | 5.27 | 8.742 | 0.059 |
| 81 | 5.83 | 6.923 | 0.064 | 0.80 | 5.610 | 0.020 | 5.60 | 9.063 | 0.069 |
| 82 | 4.75 | 6.436 | 0.064 | 0.80 | 5.599 | 0.019 | 3.70 | 7.578 | 0.047 |
| 83 | 5.48 | 6.814 | 0.060 | 0.81 | 5.606 | 0.018 | 5.45 | 8.953 | 0.069 |
| 84 | 8.70 | 7.592 | 0.059 | 0.81 | 5.623 | 0.020 | 4.47 | 8.119 | 0.034 |
| 85 | 6.46 | 7.043 | 0.064 | 0.81 | 5.581 | 0.019 | 4.98 | 8.576 | 0.040 |
| 86 | 5.37 | 6.776 | 0.063 | 0.82 | 5.617 | 0.018 | 5.53 | 8.996 | 0.063 |
| 87 | 4.67 | 6.473 | 0.041 | 0.82 | 5.626 | 0.018 | 4.14 | 7.947 | 0.056 |
| 88 | 4.89 | 6.505 | 0.091 | 0.82 | 5.580 | 0.021 | 3.87 | 7.728 | 0.051 |
| 89 | 4.47 | 6.256 | 0.058 | 0.83 | 5.664 | 0.020 | 2.07 | 6.375 | 0.038 |
| 90 | 4.65 | 6.514 | 0.059 | 0.83 | 5.666 | 0.020 | 4.74 | 8.402 | 0.040 |
| 91 | 5.42 | 6.838 | 0.066 | 0.83 | 5.666 | 0.019 | 6.23 | 9.520 | 0.054 |
| 92 | 6.00 | 6.735 | 0.062 | 0.85 | 5.684 | 0.019 | 2.98 | 7.027 | 0.033 |
| 93 | 7.68 | 7.316 | 0.063 | 0.85 | 5.684 | 0.019 | 4.26 | 8.002 | 0.060 |
| 94 | 5.26 | 6.692 | 0.107 | 0.85 | 5.646 | 0.018 | 4.64 | 8.343 | 0.038 |
| 95 | 4.61 | 6.419 | 0.060 | 0.87 | 5.689 | 0.020 | 3.08 | 7.203 | 0.033 |
| 96 | 5.13 | 6.594 | 0.064 | 0.87 | 5.696 | 0.021 | 3.91 | 7.783 | 0.051 |
| 97 | 7.46 | 7.237 | 0.059 | 0.88 | 5.696 | 0.020 | 4.28 | 7.993 | 0.049 |
| 98 | 5.18 | 6.766 | 0.060 | 0.88 | 5.704 | 0.018 | 6.25 | 9.528 | 0.056 |
| 99 | 6.04 | 6.993 | 0.064 | 0.88 | 5.689 | 0.035 | 5.54 | 9.037 | 0.050 |
| 100 | 4.16 | 6.390 | 0.061 | 0.88 | 5.693 | 0.017 | 4.15 | 8.037 | 0.048 |
| 101 | 7.53 | 7.251 | 0.059 | 0.89 | 5.699 | 0.018 | 4.33 | 8.019 | 0.042 |
| 102 | 5.67 | 6.746 | 0.062 | 0.89 | 5.676 | 0.017 | 4.10 | 7.904 | 0.051 |
| 103 | 10.98 | 8.123 | 0.064 | 0.89 | 5.642 | 0.019 | 3.04 | 7.019 | 0.129 |
| 104 | 6.53 | 7.114 | 0.064 | 0.89 | 5.634 | 0.018 | 6.27 | 9.490 | 0.201 |
| 105 | 8.33 | 7.553 | 0.059 | 0.89 | 5.631 | 0.018 | 5.44 | 8.844 | 0.056 |
| 106 | 4.85 | 6.639 | 0.059 | 0.90 | 5.633 | 0.019 | 5.24 | 8.822 | 0.074 |
| 107 | 4.48 | 6.435 | 0.068 | 0.90 | 5.645 | 0.020 | 4.39 | 8.132 | 0.044 |
| 108 | 10.60 | 8.150 | 0.062 | 0.90 | 5.625 | 0.019 | 4.66 | 8.263 | 0.042 |
| 109 | 4.85 | 6.518 | 0.067 | 0.90 | 5.637 | 0.020 | 3.61 | 7.591 | 0.049 |
| 110 | 4.60 | 6.611 | 0.058 | 0.90 | 5.642 | 0.019 | 6.08 | 9.432 | 0.071 |
| 111 | 8.18 | 7.559 | 0.062 | 0.90 | 5.642 | 0.020 | 5.35 | 8.852 | 0.058 |
| 112 | 11.20 | 8.443 | 0.061 | 0.91 | 5.647 | 0.020 | 6.35 | 9.535 | 0.060 |
| 113 | 7.03 | 7.071 | 0.059 | 0.91 | 5.644 | 0.018 | 3.30 | 7.293 | 0.046 |
| 114 | 12.01 | 8.521 | 0.061 | 0.91 | 5.677 | 0.018 | 4.33 | 8.004 | 0.049 |
| 115 | 5.61 | 6.587 | 0.059 | 0.92 | 5.681 | 0.018 | 2.44 | 6.624 | 0.040 |
| 116 | 5.57 | 6.641 | 0.051 | 0.92 | 5.684 | 0.007 | 3.19 | 7.205 | 0.038 |
| 117 | 4.61 | 6.555 | 0.058 | 0.92 | 5.690 | 0.020 | 5.28 | 8.827 | 0.071 |
| 118 | 6.09 | 6.774 | 0.063 | 0.92 | 5.688 | 0.020 | 3.09 | 7.117 | 0.047 |
| 119 | 5.61 | 6.587 | 0.059 | 0.92 | 5.675 | 0.018 | 2.44 | 6.624 | 0.049 |
| 120 | 4.58 | 6.432 | 0.111 | 0.93 | 5.690 | 0.019 | 3.57 | 7.552 | 0.044 |
| 121 | 5.61 | 6.587 | 0.068 | 0.93 | 5.688 | 0.018 | 2.44 | 6.624 | 0.041 |
| 122 | 6.01 | 6.926 | 0.063 | 0.93 | 5.693 | 0.008 | 4.96 | 8.578 | 0.134 |
| 123 | 6.35 | 6.940 | 0.061 | 0.94 | 5.703 | 0.019 | 4.18 | 7.955 | 0.055 |
| 124 | 6.56 | 7.082 | 0.060 | 0.94 | 5.700 | 0.020 | 5.64 | 9.022 | 0.046 |
| 125 | 4.35 | 6.247 | 0.062 | 0.94 | 5.699 | 0.029 | 2.25 | 6.528 | 0.036 |
| 126 | 5.85 | 6.743 | 0.059 | 0.94 | 5.686 | 0.018 | 3.50 | 7.436 | 0.042 |
| 127 | 5.87 | 6.705 | 0.059 | 0.94 | 5.686 | 0.020 | 2.76 | 6.894 | 0.053 |
| 128 | 6.53 | 7.059 | 0.059 | 0.95 | 5.695 | 0.020 | 4.38 | 8.177 | 0.035 |
| 129 | 5.99 | 6.886 | 0.060 | 0.95 | 5.701 | 0.019 | 4.77 | 8.407 | 0.037 |
| 130 | 7.71 | 7.409 | 0.066 | 0.95 | 5.695 | 0.018 | 5.30 | 8.797 | 0.074 |
| 131 | 8.80 | 7.609 | 0.063 | 0.95 | 5.706 | 0.019 | 4.23 | 7.946 | 0.149 |
| 132 | 6.12 | 6.868 | 0.071 | 0.95 | 5.707 | 0.020 | 4.33 | 8.045 | 0.046 |
| 133 | 5.15 | 6.779 | 0.063 | 0.95 | 5.705 | 0.020 | 6.29 | 9.584 | 0.052 |
| 134 | 5.33 | 6.684 | 0.060 | 0.96 | 5.707 | 0.019 | 4.41 | 8.154 | 0.049 |
| 135 | 4.34 | 6.276 | 0.059 | 0.96 | 5.709 | 0.019 | 2.92 | 7.010 | 0.047 |
| 136 | 5.75 | 6.663 | 0.064 | 0.97 | 5.712 | 0.020 | 2.16 | 6.490 | 0.042 |
| 137 | 5.99 | 6.852 | 0.062 | 0.97 | 5.716 | 0.018 | 4.19 | 7.981 | 0.055 |
| 138 | 6.34 | 6.852 | 0.058 | 0.97 | 5.697 | 0.019 | 2.99 | 7.061 | 0.046 |
| 139 | 4.16 | 6.279 | 0.063 | 0.97 | 5.697 | 0.019 | 3.10 | 7.201 | 0.044 |
| 140 | 5.65 | 6.751 | 0.062 | 0.97 | 5.710 | 0.020 | 4.19 | 7.977 | 0.051 |
| 141 | 7.79 | 7.320 | 0.059 | 0.98 | 5.727 | 0.020 | 4.16 | 7.900 | 0.048 |
| 142 | 4.52 | 6.327 | 0.059 | 0.98 | 5.723 | 0.020 | 2.52 | 6.747 | 0.045 |
| 143 | 7.49 | 7.220 | 0.059 | 0.98 | 5.724 | 0.018 | 3.76 | 7.618 | 0.053 |
| 144 | 6.34 | 7.020 | 0.059 | 0.98 | 5.727 | 0.019 | 5.57 | 8.978 | 0.042 |
| 145 | 8.42 | 7.531 | 0.062 | 0.99 | 5.728 | 0.008 | 4.89 | 8.419 | 0.044 |
| 146 | 5.63 | 6.708 | 0.061 | 0.99 | 5.730 | 0.018 | 3.70 | 7.606 | 0.053 |
| 147 | 6.20 | 7.037 | 0.059 | 1.00 | 5.741 | 0.020 | 5.74 | 9.166 | 0.063 |
| 148 | 6.28 | 6.965 | 0.063 | 1.01 | 5.729 | 0.019 | 4.76 | 8.396 | 0.149 |
| 149 | 6.25 | 6.834 | 0.061 | 1.02 | 5.748 | 0.021 | 2.99 | 7.071 | 0.038 |
| 150 | 11.50 | 8.446 | 0.068 | 1.02 | 5.752 | 0.019 | 5.56 | 8.904 | 0.066 |
| 151 | 7.15 | 7.066 | 0.059 | 1.04 | 5.754 | 0.019 | 2.71 | 6.853 | 0.045 |
| 152 | 7.37 | 7.126 | 0.062 | 1.04 | 5.756 | 0.009 | 2.78 | 6.895 | 0.047 |
| 153 | 6.10 | 6.835 | 0.060 | 1.04 | 5.763 | 0.009 | 3.24 | 7.294 | 0.046 |
| 154 | 5.27 | 6.704 | 0.131 | 1.04 | 5.720 | 0.019 | 4.44 | 8.224 | 0.118 |
| 155 | 8.36 | 7.447 | 0.062 | 1.04 | 5.754 | 0.019 | 3.57 | 7.465 | 0.037 |
| 156 | 9.16 | 7.818 | 0.059 | 1.04 | 5.760 | 0.018 | 5.60 | 8.987 | 0.063 |
| 157 | 8.25 | 7.576 | 0.062 | 1.04 | 5.763 | 0.019 | 5.26 | 8.787 | 0.177 |
| 158 | 5.86 | 6.888 | 0.062 | 1.04 | 5.768 | 0.020 | 4.14 | 8.048 | 0.053 |
| 159 | 13.65 | 8.956 | 0.060 | 1.05 | 5.770 | 0.044 | 4.47 | 8.050 | 0.048 |
| 160 | 14.95 | 9.175 | 0.083 | 1.06 | 5.772 | 0.018 | 2.61 | 6.625 | 0.189 |
| 161 | 5.13 | 6.508 | 0.063 | 1.06 | 5.774 | 0.018 | 3.11 | 7.142 | 0.038 |
| 162 | 4.30 | 6.407 | 0.059 | 1.06 | 5.773 | 0.018 | 4.76 | 8.407 | 0.046 |
| 163 | 5.36 | 6.676 | 0.058 | 1.07 | 5.775 | 0.021 | 4.49 | 8.085 | 0.039 |
| 164 | 7.00 | 7.134 | 0.059 | 1.07 | 5.775 | 0.018 | 4.47 | 8.157 | 0.049 |
| 165 | 5.10 | 6.563 | 0.062 | 1.07 | 5.774 | 0.018 | 3.58 | 7.536 | 0.051 |
| 166 | 5.46 | 6.729 | 0.061 | 1.07 | 5.813 | 0.009 | 4.62 | 8.303 | 0.042 |
| 167 | 5.08 | 6.529 | 0.059 | 1.08 | 5.817 | 0.019 | 3.24 | 7.275 | 0.048 |
| 168 | 5.56 | 6.640 | 0.091 | 1.08 | 5.815 | 0.035 | 3.05 | 7.114 | 0.044 |
| 169 | 4.53 | 6.338 | 0.064 | 1.08 | 5.793 | 0.019 | 2.68 | 6.862 | 0.042 |
| 170 | 4.59 | 6.439 | 0.062 | 1.08 | 5.817 | 0.018 | 3.67 | 7.623 | 0.046 |
| 171 | 5.06 | 6.539 | 0.059 | 1.08 | 5.813 | 0.023 | 3.39 | 7.094 | 0.042 |
| 172 | 5.86 | 6.692 | 0.061 | 1.08 | 5.813 | 0.022 | 2.49 | 6.703 | 0.149 |
| 173 | 4.56 | 6.374 | 0.059 | 1.09 | 5.826 | 0.020 | 3.52 | 7.450 | 0.050 |
| 174 | 5.47 | 6.693 | 0.064 | 1.09 | 5.826 | 0.019 | 4.60 | 8.236 | 0.046 |
| 175 | 7.00 | 7.186 | 0.038 | 1.09 | 5.826 | 0.021 | 4.47 | 8.226 | 0.038 |
| 176 | 8.11 | 7.354 | 0.063 | 1.09 | 5.828 | 0.019 | 3.54 | 7.416 | 0.043 |
| 177 | 3.94 | 6.230 | 0.062 | 1.09 | 5.827 | 0.019 | 3.68 | 7.600 | 0.052 |
| 178 | 4.76 | 6.634 | 0.059 | 1.09 | 5.830 | 0.009 | 5.77 | 9.197 | 0.066 |
| 179 | 4.41 | 6.470 | 0.064 | 1.10 | 5.831 | 0.028 | 4.88 | 8.529 | 0.058 |
| 180 | 5.32 | 6.743 | 0.060 | 1.11 | 5.772 | 0.020 | 5.73 | 9.103 | 0.055 |
| 181 | 5.65 | 6.739 | 0.062 | 1.11 | 5.768 | 0.022 | 3.95 | 7.803 | 0.052 |
| 182 | 4.67 | 6.473 | 0.060 | 1.11 | 5.770 | 0.018 | 4.14 | 7.947 | 0.054 |
| 183 | 5.07 | 6.588 | 0.063 | 1.12 | 5.772 | 0.018 | 4.14 | 7.948 | 0.052 |
| 184 | 4.23 | 6.236 | 0.058 | 1.12 | 5.773 | 0.020 | 2.66 | 6.827 | 0.040 |
| 185 | 5.29 | 6.514 | 0.069 | 1.12 | 5.776 | 0.018 | 2.56 | 6.728 | 0.041 |
| 186 | 5.43 | 6.635 | 0.064 | 1.13 | 5.758 | 0.024 | 3.35 | 7.355 | 0.054 |
| 187 | 5.56 | 6.640 | 0.062 | 1.14 | 5.776 | 0.018 | 3.05 | 7.811 | 0.038 |
| 188 | 4.56 | 6.374 | 0.059 | 1.15 | 5.786 | 0.010 | 3.52 | 7.450 | 0.051 |
| 189 | 5.39 | 6.844 | 0.119 | 1.15 | 5.761 | 0.022 | 6.07 | 9.435 | 0.087 |
| 190 | 5.90 | 6.803 | 0.061 | 1.15 | 5.797 | 0.016 | 3.81 | 7.702 | 0.059 |
| 191 | 6.30 | 6.959 | 0.063 | 1.15 | 5.827 | 0.016 | 4.64 | 8.302 | 0.044 |
| 192 | 6.86 | 7.169 | 0.061 | 1.15 | 5.833 | 0.012 | 5.13 | 8.690 | 0.055 |
| 193 | 4.74 | 6.436 | 0.059 | 1.15 | 5.827 | 0.016 | 3.14 | 7.215 | 0.079 |
| 194 | 6.14 | 6.828 | 0.059 | 1.16 | 5.834 | 0.016 | 3.55 | 7.073 | 0.047 |
| 195 | 5.74 | 6.766 | 0.063 | 1.16 | 5.830 | 0.016 | 4.03 | 7.858 | 0.053 |
| 196 | 5.33 | 6.781 | 0.063 | 1.16 | 5.831 | 0.015 | 4.89 | 8.600 | 0.160 |
| 197 | 5.40 | 6.654 | 0.058 | 1.16 | 5.833 | 0.016 | 3.64 | 7.582 | 0.059 |
| 198 | 6.71 | 6.898 | 0.060 | 1.17 | 5.833 | 0.017 | 2.29 | 6.522 | 0.049 |
| 199 | 8.15 | 7.389 | 0.059 | 1.18 | 5.832 | 0.007 | 3.29 | 7.283 | 0.041 |
| 200 | 13.20 | 8.867 | 0.066 | 1.18 | 5.844 | 0.015 | 5.16 | 8.555 | 0.052 |
| 201 | 4.86 | 6.516 | 0.059 | 1.19 | 5.850 | 0.016 | 4.09 | 6.899 | 0.053 |
| 202 | 5.69 | 6.674 | 0.057 | 1.20 | 5.855 | 0.017 | 3.30 | 7.275 | 0.047 |
| 203 | 4.92 | 6.595 | 0.060 | 1.20 | 5.858 | 0.016 | 5.09 | 9.264 | 0.189 |
| 204 | 5.63 | 6.999 | 0.059 | 1.22 | 5.864 | 0.016 | 7.05 | 10.192 | 0.205 |
| 205 | 5.78 | 6.811 | 0.065 | 1.23 | 5.875 | 0.016 | 4.36 | 8.119 | 0.045 |
| 206 | 5.05 | 6.617 | 0.059 | 1.23 | 5.882 | 0.025 | 4.57 | 8.928 | 0.051 |
| 207 | 4.71 | 6.567 | 0.066 | 1.24 | 5.876 | 0.016 | 5.20 | 8.752 | 0.065 |
| 208 | 5.28 | 6.693 | 0.059 | 1.24 | 5.888 | 0.024 | 4.65 | 8.342 | 0.046 |
| 209 | 4.00 | 6.566 | 0.060 | 1.24 | 5.851 | 0.016 | 7.83 | 10.748 | 0.206 |
| 210 | 5.17 | 6.609 | 0.058 | 1.24 | 5.891 | 0.025 | 4.03 | 7.865 | 0.070 |
| 211 | 5.98 | 6.903 | 0.041 | 1.24 | 5.876 | 0.016 | 4.83 | 8.473 | 0.044 |
| 212 | 5.38 | 6.735 | 0.062 | 1.26 | 5.899 | 0.015 | 5.03 | 8.092 | 0.046 |
| 213 | 4.41 | 6.374 | 0.060 | 1.26 | 5.893 | 0.018 | 3.61 | 7.566 | 0.071 |
| 214 | 5.81 | 6.918 | 0.064 | 1.28 | 5.900 | 0.017 | 5.97 | 9.307 | 0.070 |
| 215 | 5.16 | 6.552 | 0.107 | 1.28 | 5.914 | 0.016 | 3.43 | 7.399 | 0.112 |
| 216 | 5.59 | 6.598 | 0.060 | 1.28 | 5.893 | 0.018 | 2.45 | 6.654 | 0.047 |
| 217 | 4.93 | 6.606 | 0.062 | 1.28 | 5.898 | 0.015 | 4.82 | 8.471 | 0.042 |
| 218 | 9.90 | 7.955 | 0.060 | 1.28 | 5.915 | 0.020 | 5.03 | 8.121 | 0.041 |
| 219 | 5.33 | 6.721 | 0.063 | 1.30 | 5.913 | 0.015 | 4.88 | 8.512 | 0.047 |
| 220 | 6.45 | 7.142 | 0.057 | 1.39 | 5.970 | 0.016 | 6.51 | 9.716 | 0.284 |
| 221 | 5.47 | 6.698 | 0.058 | 1.40 | 5.978 | 0.030 | 3.70 | 7.254 | 0.056 |
| 222 | 6.02 | 6.959 | 0.059 | 1.45 | 6.024 | 0.026 | 5.66 | 9.308 | 0.048 |
| 223 | 4.99 | 6.631 | 0.066 | 1.45 | 6.014 | 0.029 | 5.11 | 8.967 | 0.060 |
| 224 | 12.81 | 8.724 | 0.059 | 1.45 | 5.997 | 0.030 | 4.27 | 7.930 | 0.051 |
| 225 | 5.81 | 6.883 | 0.065 | 1.46 | 6.018 | 0.027 | 5.54 | 7.977 | 0.151 |
| 226 | 5.84 | 6.785 | 0.065 | 1.48 | 6.025 | 0.032 | 3.94 | 7.786 | 0.068 |
| 227 | 7.37 | 7.126 | 0.063 | 1.48 | 6.025 | 0.026 | 2.78 | 6.290 | 0.051 |
| 228 | 6.10 | 6.835 | 0.065 | 1.52 | 6.051 | 0.029 | 3.24 | 7.294 | 0.053 |
| 229 | 5.27 | 6.704 | 0.111 | 1.52 | 6.036 | 0.029 | 4.44 | 8.224 | 0.032 |
| 230 | 8.36 | 7.447 | 0.063 | 1.52 | 6.051 | 0.012 | 3.57 | 7.465 | 0.031 |
| 231 | 9.16 | 7.818 | 0.064 | 1.53 | 6.051 | 0.029 | 5.60 | 8.987 | 0.044 |
| 232 | 12.53 | 8.502 | 0.060 | 1.55 | 6.067 | 0.027 | 2.45 | 6.546 | 0.110 |
| 233 | 8.10 | 7.507 | 0.060 | 1.55 | 6.075 | 0.030 | 5.37 | 8.825 | 0.046 |
| 234 | 6.75 | 6.981 | 0.059 | 1.56 | 6.083 | 0.030 | 3.28 | 7.267 | 0.032 |
| 235 | 6.18 | 7.051 | 0.062 | 1.58 | 6.090 | 0.003 | 6.05 | 9.396 | 0.155 |
| 236 | 5.97 | 6.794 | 0.059 | 1.61 | 6.112 | 0.029 | 3.58 | 7.512 | 0.037 |
| 237 | 7.01 | 7.141 | 0.059 | 1.66 | 6.135 | 0.028 | 4.16 | 7.958 | 0.041 |
| 238 | 6.80 | 7.052 | 0.067 | 0.70 | 5.716 | 0.013 | 4.27 | 7.991 | 0.049 |
| 239 | 4.53 | 6.702 | 0.060 | 0.80 | 5.423 | 0.013 | 7.54 | 10.538 | 0.105 |
| 240 | 9.66 | 7.825 | 0.065 | 0.85 | 5.525 | 0.009 | 4.02 | 7.767 | 0.046 |
| 241 | 9.16 | 7.620 | 0.063 | 1.09 | 5.740 | 0.033 | 3.24 | 7.173 | 0.029 |
| 242 | 6.80 | 7.052 | 0.064 | 1.38 | 5.793 | 0.028 | 4.27 | 7.991 | 0.045 |

2 Evaluation study of SELF model based on EM metabolite sensor

2.1 Comparison and analysis of different model for CVDs risk assessment

**Tab. S7** Comparison of cardiovascular and cerebrovascular risk assessment models

| **Assessment Models** | **Research Population** | **Publish time** | **Risk factors** | **Assessment endpoints** | **Research Guides& Used** |
| --- | --- | --- | --- | --- | --- |
| Framingham-CHD | European Americans, 2489 men and 2856 women without CHD, aged 30 to 74 years, with 12 years of follow-up | 1998 | Age, sex, TC, LDL-C, HDL-C, blood pressure, diabetes and smoking | 10-year risk: angina, MI and CHD death | Framingham |
|  |  |  |  |  |  |
| ATP-Ⅲ | European Americans, 2489 men and 2856 women without CHD, aged 30 to 74 years, with 12 years of follow-up | 2001 | Age, sex, TC, HDL-C, blood pressure, blood pressure treatment status and smoking | 10-year risk: MI and CHD death | Framingham & 2001 NCEP ATP-III |
| EURO-SCORE | European population of 12 countries, 80,080 men and 117,098 women without CVD | 2003 | Age, sex, TC, HDL-C, blood pressure and smoking | 10-year risk: CHD death, stroke death and coronary revascularization | 12 Cohorts & 2011 ESC/EAS Guidelines for the Management of Dyslipidemia |
| Lloyd-Jones/ Framingham algorithm | European Americans, 3564 men and 4362 women without CVD, age 50 years, with 111,777 follow-up | 2006 | Age, gender, TC, blood pressure, diabetes, current smoking and BMI | Lifetime risk: MI, coronary ischemia, CHD death, angina pectoris, ischemic stroke, intermittent claudication, death from other cardiovascular causes | Framingham & 2013 IAS Global Recommendations for Dyslipidemia Management |
| ICVD | Chinese, 4890 males and 5013 females without CHD and stroke, aged 35-59 years, with a mean follow-up of 15.1 years | 2006 | Age, sex, blood pressure, TC, BMI, smoking and diabetes | 10-year risk: MI, stroke and CVD death | USA-PRC & 2010 Chinese Expert Consensus on Primary Prevention of Cardiovascular Disease |
| Reynolds-women | European Americans, 24558 healthy women, age ≥45 years, 10.2 years follow-up | 2007 | Age, TC, HDL-C, hs-CRP, blood pressure, HbA1c, smoking and family history of early onset MI | 10-year risk: MI, CHD death, stroke, stroke death and coronary revascularization | Women’s Health Study |
| Reynolds-men | European Americans, free of diabetes and CVD, 10,724 men, age ≥50 years, 10.8 years of follow-up | 2008 | Age, TC, HDL-C, hs-CRP, blood pressure, smoking and family history of early onset MI | 10-year risk: MI, CHD death, stroke, stroke death and coronary revascularization | Physicians Health Study |
| Framingham-Global | European Americans, 4419 men and 4522 women without CVD, age 30-74 years, 12 years follow-up | 2008 | Age, sex, TC, HDL-C, blood pressure, blood pressure treatment status, diabetes and smoking | 10-year risk: MI, CHD death, stroke, stroke death and heart failure | Framingham |
| QRISK2 | British (96.5%), no history of CVD or statin use, 1136,761 men and 1149,054 women, age 35-74 years, 16 million years of follow-up | 2008 | Age, sex, blood pressure, blood pressure treatment status, smoking, TC/ HDL-C, family history of early-onset CHD, BMI, social deprivation, rheumatoid arthritis, atrial fibrillation, chronic kidney disease | 10-year risk: MI, angina, stroke and TIA | QRESEARCH & 2014 UK NICE guidelines on lipid management |
| WHO /ISH | — | 2008 | Age, gender, blood pressure, smoking, TC and diabetes | 10-year risk: fatal MI or stroke, non-fatal MI or stroke | — |
| Pooled Cohort Risk Equations | European and African Americans, healthy individuals, 10,745 men and 13,881 women, age 40 to 79 years, follow-up >12 years | 2013 | Age, sex, TC, HDL-C, blood pressure, blood pressure treatment status, diabetes and current smoking status | 10-year risk: CHD death, fatal or non-fatal stroke | ARIC，CARDIA，CHS，Framingham & 2013 ACC/AHA Guidelines for Cholesterol Control to Reduce ASCVD Risk |
| China PAR | Chinese, adults aged 35-59 years, 27,000 cohort population, mean follow-up time 12.3 years | 2016 | Age, systolic blood pressure, whether taking antihypertensive drugs, total cholesterol, HDL cholesterol, smoking and diabetes, waist circumference, north-south, urban-rural and family history of ASCVD | 10-year risk: CHD, ASCVD, acute myocardial infarction, death from coronary artery disease and fatal and non-fatal stroke | China MUCA; Framingham & 2013 ACC/AHA Guidelines for Cholesterol Control to Reduce ASCVD Risk |

2.2 Analysis on baseline data and establishment of SELF model

Individuals from urban and rural communities in ASSTTH were considered for inclusion. Written informed consent was obtained from each of the involved inpatients. We recorded information on participants' sociodemographic characteristics, risk factors, medication use and cardiac investigations for incident cardiovascular disease. SPSS 20.0 statistical software was used for analysis. All variants were screened by univariate analysis to determine the independent risk factors. Based on the results of the clinical sample tests, the history of disease from these patients was collected. Inpatients were divided into very low risk, 1; low risk, 2; medium risk, 3; high risk, 4; and very high risk, 5. According to their inpatient cases and evaluation results of the standard scale, the weight of risk factors was established by the analysis of the risk 3-5 inpatients. The grading standard of the model was established by univariate analysis and conditional logistic regression and optimized by the analysis of risk 1-2 inpatients. Unique samples with different results were used to optimize the aggregation factor again. Comparison with other models to validate the assessment effect. Establishment and optimization of a risk assessment model of CVDs

Disease risk assessment was a technique used to study the quantitative dependence and pattern of disease-causing risk factors and the incidence of specific diseases and was generally considered to be the basis and core function of health management. CVDs have a slow onset and relatively long latency period, and early intervention by studying individual risk factors for CVDs was currently recognized as the most effective means of reducing CVD mortality [4].

Many studies have developed assessment tools based on various cardiovascular disease prediction models. Tab. S7 shows the traditional predictive tools for risk assessment of CVDs based on long-term follow-up of large samples [5]. Treating patients who were assessed as high risk with multivariable prediction equations was also more effective than treating patients with high levels of single risk. Therefore, it was obviously insufficient to detect only biochemical metabolic indexes. Basic information about patients and clinical diagnoses needs to be comprehensively analyzed. The EM metabolite sensor was used to establish the SELF model based on the EMPS-based detection and medical history of each patient. Baseline data were shown in Tab. S8.

**Tab. S8** Baseline dates of different groups patients

| Factors | Baseline dates of different risk patients | | | | | |
| --- | --- | --- | --- | --- | --- | --- |
|  | Testing group (n=80) | | Training group (n=120) | | | verification group (n=42) |
|  | Level 1 (n=40) | Level 2 (n=40) | Level 3 (n=40) | Level 4(n=40) | Level 5 (n=40) |  |
| Gender | 0.48±0.51 | 0.6±0.5 | 0.65±0.48 | 0.65±0.48 | 0.53±0.51 | 0.45±0.5 |
| Age | 47.23±14.14 | 60.28±11.05 | 58.6±15.01 | 63.75±11.97 | 69.28±11.69 | 66.53±10.91 |
| Systolic blood pressure | 120.75±12.24 | 135.88±18.-47 | 140.05±25.37 | 142.08±21.41 | 128.23±19.35 | 145.23±21.38 |
| BMI | 22.62±2.61 | 24.43±3.05 | 25.57±3.87 | 24.58±3.42 | 24.02±3.63 | 25.39±3.43 |
| Glucose | 5.2±0.66 | 5.78±1.11 | 6.28±2.03 | 6.88±2.26 | 5.67±1.55 | 6.58±2.1 |
| Total Chol | 4.38±0.96 | 4.36±1.04 | 4.41±1.15 | 4.04±1.11 | 3.92±0.94 | 4.51±1.22 |
| Lactate | 0.88±0.23 | 0.94±0.28 | 0.95±0.31 | 0.98±0.28 | 0.91±0.23 | 0.95±0.26 |
| History of hypertension | 0±0 | 0.38±0.49 | 0.68±0.47 | 0.78±0.42 | 0.88±0.33 | 0.5±0.51 |
| History of Heart attack | 0±0 | 0±0 | 0.03±0.16 | 0.15±0.36 | 0.28±0.45 | 0.1±0.3 |
| History of Cardiac insufficiency | 0±0 | 0±0 | 0.03±0.16 | 0.05±0.22 | 0.03±0.16 | 0±0 |
| Cerebral infarction history | 0±0 | 0.03±0.16 | 0.08±0.27 | 0.15±0.36 | 0.23±0.42 | 0.1±0.3 |
| History of diabetes | 0.08±0.47 | 0.1±0.3 | 0.18±0.38 | 0.13±0.33 | 0.33±0.47 | 0.55±1.65 |
| Smoking history | 0.03±0.16 | 0.33±0.47 | 0.13±0.33 | 0.15±0.36 | 0.18±0.38 | 0.15±0.36 |

The risk factors with statistical significance in univariate analysis were analyzed by the Cox proportional risk regression model of stepwise regression. The results of the univariate analysis were shown in the Tab.S9. The six factors of age, blood pressure, BMI, Glu and Chol disease history had high weights in the risk assessment for CVDs. Although lactic acid could not be used as an independent risk factor, the observed increase and decrease in the lactic acid value was characteristic of the collective metabolic level. This value was used as an auxiliary reference factor, and the impact on blood pressure, blood Glu and blood lipid factors was used as an additional score in the model [6]. Therefore, based on these six risk factors, we established a preliminary SELE model for the risk assessment of CVDs. Multiple factor analysis of the CVD model was shown in Tab. S10.

**Tab. S9** Single factor analysis of CVDs model

| Factors | Variable | Coefficients | Standard Error | | t Stat | P-value | Lower 95% | | Upper 95% |
| --- | --- | --- | --- | --- | --- | --- | --- | --- | --- |
| Age | Intercept | 0.7103 | 0.3768 | | 1.8847 | 0.0609 | -0.0329 | | 1.4534 |
|  | X Variable 1 | 0.0416 | 0.0061 | | 6.8017 | 0.0000 | 0.0296 | | 0.0537 |
| Blood Pressure | Intercept | 1.6790 | 0.6268 | | 2.6789 | 0.0080 | 0.4430 | | 2.9150 |
|  | X Variable 1 | 0.0114 | 0.0046 | | 2.4570 | 0.0149 | 0.0023 | | 0.0206 |
| BMI | Intercept | 1.8102 | 0.7010 | | 2.5822 | 0.0105 | 0.4277 | | 3.1927 |
|  | X Variable 1 | 0.0573 | 0.0286 | | 2.0023 | 0.0466 | 0.0009 | | 0.1138 |
| Glucose | Intercept | 2.1854 | 0.3533 | | 6.1862 | 0.0000 | 1.4887 | | 2.8820 |
|  | X Variable 1 | 0.1702 | 0.0570 | | 2.9877 | 0.0032 | 0.0579 | | 0.2825 |
| Total Chol | Intercept | 4.0485 | 0.4074 | | 9.9376 | 0.0000 | 3.2451 | | 4.8519 |
|  | X Variable 1 | -0.2010 | 0.0937 | | -2.1462 | 0.0331 | -0.3857 | | -0.0163 |
| Lactate | Intercept | 2.9363 | 0.3593 | | 8.1725 | 0.0000 | 2.2278 | | 3.6448 |
|  | X Variable 1 | 0.2831 | 0.3707 | | 0.7637 | 0.4459 | -0.4479 | | 1.0142 |
| History of hypertension | Intercept | 2.2717 | 0.1160 | | 19.5807 | 0.0000 | 2.0429 | | 2.5005 |
|  | X Variable 1 | 1.7190 | 0.1579 | | 10.8878 | 0.0000 | 1.4077 | | 2.0303 |
| History of Heart attack | Intercept | 3.0604 | 0.0989 | | 30.9388 | 0.0000 | 2.8654 | | 3.2555 |
|  | X Variable 1 | 1.5507 | 0.3297 | | 4.7028 | 0.0000 | 0.9004 | | 2.2009 |
| History of Cardiac insufficiency | Intercept | 3.1786 | 0.0999 | | 31.8095 | 0.0000 | 2.9815 | | 3.3756 |
|  | X Variable 1 | 1.0714 | 0.7066 | | 1.5164 | 0.1310 | -0.3220 | | 2.4648 |
| Cerebral infarction history | Intercept | 3.0773 | 0.1007 | | 30.5628 | 0.0000 | 2.8788 | | 3.2759 |
|  | X Variable 1 | 1.2911 | 0.3267 | | 3.9521 | 0.0001 | 0.6469 | | 1.9353 |
| History of diabetes | Intercept | 3.0900 | 0.1048 | | 29.4795 | 0.0000 | 2.8833 | | 3.2967 |
|  | X Variable 1 | 0.6873 | 0.2405 | | 2.8583 | 0.0047 | 0.2131 | | 1.1616 |
| Smoking history | Intercept | 3.1607 | 0.1083 | | 29.1762 | 0.0000 | 2.9471 | | 3.3743 |
|  | X Variable 1 | 0.2455 | 0.2708 | | 0.9066 | 0.3657 | -0.2885 | | 0.7796 |
| * p<0.05 ** p<0.01 | | | |  | | | |  |  |

**Tab. S10** Multiple factor analysis of CVDs model

| Factors | Coefficients | Standard Error | t Stat | | P-value | Lower 95% | Upper 95% | |
| --- | --- | --- | --- | --- | --- | --- | --- | --- |
| Intercept | -0.0636 | 0.7774 | -0.0818 | | 0.9349 | -1.5973 | 1.4700 | |
| Age | 0.0193 | 0.0053 | 3.6581 | | 0.0003 | 0.0089 | 0.0297 | |
| Systolic blood pressure | 0.0029 | 0.0035 | 0.8410 | | 0.4014 | -0.0040 | 0.0099 | |
| BMI | 0.0284 | 0.0219 | 1.2936 | | 0.1974 | -0.0149 | 0.0716 | |
| Glucose | 0.0401 | 0.0410 | 0.9770 | | 0.3298 | -0.0408 | 0.1209 | |
| Total Chol | -0.0580 | 0.0662 | -0.8771 | | 0.3816 | -0.1886 | 0.0725 | |
| Lactate | -0.0363 | 0.2614 | -0.1390 | | 0.8896 | -0.5520 | 0.4793 | |
| History of hypertension | 1.3979 | 0.1565 | 8.9343 | | 0.0000 | 1.0893 | 1.7066 | |
| History of Heart attack | 0.7238 | 0.2492 | 2.9044 | | 0.0041 | 0.2322 | 1.2155 | |
| History of Cardiac insufficiency | 1.1401 | 0.4997 | 2.2813 | | 0.0237 | 0.1542 | 2.1259 | |
| Cerebral infarction history | 0.7585 | 0.2403 | 3.1562 | | 0.0019 | 0.2844 | 1.2326 | |
| History of diabetes | 0.7020 | 0.1719 | 4.0839 | | 0.0001 | 0.3629 | 1.0411 | |
| Smoking history | 0.2612 | 0.1896 | 1.3774 | | 0.1700 | -0.1129 | 0.6352 | |
| * p<0.05 ** p<0.01 | | | |  | | | |  |

The fitting analysis results of the modeling were shown in the table S11.

**Tab. S11** Multiple Regression analysis of CVDs model

| Factors | Coefficients | Standard Error | t Stat | | P-value | Lower 95% | Upper 95% | |
| --- | --- | --- | --- | --- | --- | --- | --- | --- |
| Intercept | -1.0674 | 0.1091 | -9.7807 | | 0 | -1.2826 | -0.8521 | |
| History | 0.7716 | 0.0246 | 31.3618 | | 0 | 0.723 | 0.8201 | |
| Age | 0.0873 | 0.0202 | 4.3266 | | 0 | 0.0475 | 0.1271 | |
| Systolic blood pressure | 0.5225 | 0.0205 | 25.4805 | | 0 | 0.4821 | 0.563 | |
| BMI | 0.0055 | 0.018 | -0.3031 | | 0.7622 | -0.041 | 0.0301 | |
| Glucose | 0.0571 | 0.0277 | 2.0573 | | 0.041 | 0.0024 | 0.1118 | |
| Total Chol | 0.0527 | 0.0217 | 2.4269 | | 0.0161 | 0.0099 | 0.0954 | |
| * p<0.05 ** p<0.01 | | | |  | | | |  |

Based on the above classification criteria, we also obtained a more comprehensive classification of medical history factors to optimize and correct the index factor classification [7]. Hypertension, diabetes, smoking, cardiac insurance, heart attack and cerebral information were high-risk factor items. Different diseases corresponded to different grades. In addition, we considered the number of risk factor items to calibrate the criteria of medical history risk classification. The classification standard optimization SELF model was shown in Tab. S12.

**Tab. S12** Hazard factor classification

| \| Factors Score \| \| 1 \| 2 \| 3 \| 4 \| 5 \| \| --- \| --- \| --- \| --- \| --- \| --- \| --- \| \| Age \| \| <41 \| 41-50 \| 51-55 \| 56-60 \| >60 \| \| BMI \| \| <24 \| 24-25.5 \| 25.6-26.5 \| 26.6-28 \| >28 \| \| Blood Pressure (mmHg) \| Value \| <120 \| 121-130 \| 131-140 \| 141-160 \| >160 \| \| High Lac \| Value+2 \| Value+4 \| Value+6 \| Value+8 \| Value+10 \| \| Glu (mM) \| Value \| <6.1 \| 6.1-7.1 \| 7.2-8.8 \| 8.9-11.1 \| >11.1 \| \| High Lac \| Value+0.02 \| Value+0.04 \| Value+0.06 \| Value+0.08 \| Value+0.1 \| \| Chol (mM) \| Value \| <4.5 \| 4.5-5 \| 5.1-5.4 \| 5.5-6 \| >6 \| \| High Lac \| Value+0.02 \| Value+0.04 \| Value+0.06 \| Value+0.08 \| Value+0.1 \| \| Medical History \| Hypertension \| No \| No \| Yes \| No \| No \| \| Diabetes \| No \| No \| Yes \| No \| No \| \| Smoking \| No \| Yes \| No \| No \| No \| \| Cardiac insufficiency \| No \| No \| Yes \| No \| No \| \| Heart attack \| No \| No \| No \| Yes \| No \| \| Cerebral infarction \| No \| No \| No \| Yes \| No \| \| Hazardous items \| 0 \| 1 \| 2 \| 3 \| >3 \| \| Summation level \| 0 \| 1-2 \| 3-6 \| 7-10 \| >10 \| |
| --- | --- | --- | --- | --- | --- | --- | --- | --- | --- | --- | --- | --- | --- | --- | --- | --- | --- | --- | --- | --- | --- | --- | --- | --- | --- | --- | --- | --- | --- | --- | --- | --- | --- | --- | --- | --- | --- | --- | --- | --- | --- | --- | --- | --- | --- | --- | --- | --- | --- | --- | --- | --- | --- | --- | --- | --- | --- | --- | --- | --- | --- | --- | --- | --- | --- | --- | --- | --- | --- | --- | --- | --- | --- | --- | --- | --- | --- | --- | --- | --- | --- | --- | --- | --- | --- | --- | --- | --- | --- | --- | --- | --- | --- | --- | --- | --- | --- | --- | --- | --- | --- | --- | --- | --- | --- | --- | --- | --- | --- |
|  |

After improving the criteria of the index risk classification and medical history risk classification, we calibrated the level of the SELF model in the risk assessment of CVDs and compared it with those of ASCVDs, ICVDs and FRAM. The detailed data fitting results were shown in Tab. S13. The obtained regression equation of the SELF model was risk score = 0.7716 * medical history + 0.0873 * age + 0.5225 * high pressure level + 0.0055 * BMI level + 0.0571 * glucose level+ 0.0527 * cholesterol grade - 1.0674, and the fitted correlation was 0.967. The risk score of cardiovascular and cerebrovascular diseases could be calculated by this equation, and the risk level could be determined by the interval to which the score belongs.

**Tab. S13** The fitting analysis results of the SELF model

| Multiple R | Analysis | df | SS | MS | F | Significance F |
| --- | --- | --- | --- | --- | --- | --- |
| 0.967 | Regression | 6 | 374.1242 | 62.354 | 465.0795 | 0 |
|  | Residual | 193 | 25.8758 | 0.1341 | 0 | 0 |
|  | Total | 199 | 400 | 0 | 0 | 0 |

2.3 Clinical analysis results and information

The clinical analysis results and information were shown in Tab. S14-S17, the CV of the coincidence rate of the SELF model was 94.6% compared with the results of clinical diagnosis. FRAM, a classic model for the risk assessment of CVDs, takes Europeans as the research subjects. Due to the differences in physique and region, the model overestimates the incidence risk of Chinese patients, and the CV of the coincidence rate was 62.7%. Although the ICVD model was a classic evaluation model for Chinese people, changes in living environment have affected the disease risk evaluation of the model to a certain extent because the model has been established for some time, and the CV of coincidence rate was 32.7%. In the ASCVD model, the overall risk assessment of patients was low. Because the model was for the risk assessment of ASCVDs in a limited age group, it led to a deviation in the evaluation results, and the CV of the coincidence rate was 94.6%[8, 9].

**Tab. S14** Comparison of the results between the SELF model, clinical diagnosis and other models

(Results: number of enrolled samples, total: 44 groups)

| Level | | Extremely low | Low | Medium | High | Extremely high | Consistency |
| --- | --- | --- | --- | --- | --- | --- | --- |
| Clinical Diagnosis | Results | 8 | 10 | 7 | 11 | 6 | 100% |
| SELF MODEL | Standard | <1.61 | 1.61-2.79 | 2.80-3.52 | 3.53-4.23 | >4.23 | 94.6% |
|  | Results | 8 | 9 | 7 | 12 | 6 |  |
|  | Compliance rate | 100% | 90.0% | 1005 | 90.9% | 100% |  |
|  | RCV | 4.3% | 6.2% | 3.3% | 5.7% | 3.0% |  |
| ASCVDs MODEL | Standard | <2.6% | 2.6%-5% | 5.1%-10% | 10.1%-20% | >20% |  |
|  | Results | 12 | 12 | 9 | 6 | 3 |  |
|  | Compliance rate | 150.0% | 120.0% | 128.6% | 54.5% | 50.0% | 54.8% |
|  | RCV | 5.2% | 6.3% | 6.4% | 3.6% | 2.3% |  |
| ICVDs MODEL | Standard | <0.6% | 0.6%-5% | 5.1%-15% | 15.1%-35% | >35% |  |
|  | Results | 9 | 13 | 9 | 7 | 4 |  |
|  | Compliance rate | 112.5% | 130.0% | 128.6% | 63.6% | 66.7% | 67.3% |
|  | RCV | 4.1% | 3.4% | 6.3% | 6.7% | 5.2% |  |
| FRAM MODEL | Standard | <5% | 5%-10% | 11%-20% | 21%-30% | >30% |  |
|  | Results | 3 | 6 | 6 | 15 | 12 |  |
|  | Compliance rate | 37.5% | 60.0% | 85.7% | 136.4% | 200% | 37.3% |
|  | RCV | 3.3% | 5.3% | 4.4% | 2.7% | 3.9% |  |

**Tab. S15** Consistency check between clinical diagnosis and different model

| Consistency Check | | | | | | |
| --- | --- | --- | --- | --- | --- | --- |
|  | Kappa Value | Standard Error | z value | p value | Standard Error | 95% CI |
| Clinical Diagnosis & SELF model | 1.000 | 0.141 | 7.071 | 0.000** | 0.000 | 1.000 ~ 1.000 |
| Clinical Diagnosis & ICVD model | 0.869 | 0.140 | 6.196 | 0.000** | 0.073 | 0.726 ~ 1.012 |
| Clinical Diagnosis & FRAM model | 0.798 | 0.139 | 5.762 | 0.000** | 0.084 | 0.634 ~ 0.963 |
| SELF model & ICVD model | 0.869 | 0.140 | 6.196 | 0.000** | 0.073 | 0.726 ~ 1.012 |
| SELF model & FRAM model | 0.798 | 0.139 | 5.762 | 0.000** | 0.084 | 0.634 ~ 0.963 |
| * p<0.05 ** p<0.01 |  |  |  |  |  |  |

**Tab. S16** Clinical analysis results and information of testing and training groups

| Number | Clinical Diagnosis | Gender | Age | Systolic blood pressure | BMI | Glucose | Total Chol | Lactate | History of hypertension | History of Heart attack | History of Cardiac insufficiency | Cerebral infarction history | History of diabetes | Smoking history |
| --- | --- | --- | --- | --- | --- | --- | --- | --- | --- | --- | --- | --- | --- | --- |
| 1 | 1 | 0 | 29 | 110 | 25.22 | 5.11 | 5.45 | 0.62 | 0 | 0 | 0 | 0 | 0 | 0 |
| 2 | 1 | 1 | 31 | 116 | 26.37 | 5.51 | 3.66 | 0.99 | 0 | 0 | 0 | 0 | 0 | 0 |
| 3 | 1 | 1 | 60 | 129 | 24.80 | 4.74 | 5.11 | 0.98 | 0 | 0 | 0 | 0 | 0 | 0 |
| 4 | 1 | 1 | 52 | 122 | 23.79 | 6.85 | 2.91 | 0.73 | 0 | 0 | 0 | 0 | 0 | 0 |
| 5 | 1 | 0 | 34 | 103 | 21.03 | 4.54 | 3.02 | 1.05 | 0 | 0 | 0 | 0 | 0 | 0 |
| 6 | 1 | 1 | 65 | 112 | 24.15 | 7.98 | 3.26 | 1.18 | 0 | 0 | 0 | 0 | 0 | 0 |
| 7 | 1 | 1 | 40 | 118 | 20.76 | 4.62 | 4.12 | 1.24 | 0 | 0 | 0 | 0 | 0 | 0 |
| 8 | 1 | 1 | 53 | 107 | 24.64 | 4.82 | 3.31 | 1.00 | 0 | 0 | 0 | 0 | 0 | 0 |
| 9 | 1 | 0 | 57 | 130 | 23.67 | 4.22 | 4.76 | 0.90 | 0 | 0 | 0 | 0 | 0 | 0 |
| 10 | 1 | 0 | 46 | 111 | 24.01 | 4.78 | 5.65 | 0.65 | 0 | 0 | 0 | 0 | 0 | 0 |
| 11 | 1 | 1 | 68 | 105 | 23.83 | 6.31 | 4.44 | 1.04 | 0 | 0 | 0 | 0 | 0 | 0 |
| 12 | 2 | 1 | 54 | 134 | 23.02 | 5.10 | 5.75 | 1.06 | 0 | 0 | 0 | 0 | 0 | 0 |
| 13 | 2 | 1 | 44 | 104 | 26.23 | 4.61 | 5.28 | 1.09 | 0 | 1 | 0 | 0 | 0 | 0 |
| 14 | 1 | 0 | 62 | 116 | 20.90 | 5.00 | 5.52 | 0.76 | 0 | 0 | 0 | 0 | 0 | 0 |
| 15 | 1 | 0 | 51 | 102 | 21.50 | 4.54 | 5.71 | 0.64 | 0 | 0 | 0 | 0 | 0 | 0 |
| 16 | 2 | 0 | 55 | 131 | 23.31 | 5.69 | 3.30 | 0.57 | 0 | 0 | 0 | 0 | 0 | 0 |
| 17 | 1 | 0 | 32 | 128 | 23.64 | 4.78 | 3.66 | 0.56 | 0 | 0 | 0 | 0 | 0 | 0 |
| 18 | 2 | 0 | 50 | 132 | 23.75 | 4.57 | 5.24 | 0.62 | 0 | 0 | 0 | 0 | 0 | 0 |
| 19 | 1 | 0 | 63 | 116 | 20.96 | 4.70 | 4.71 | 0.78 | 0 | 0 | 0 | 0 | 0 | 0 |
| 20 | 2 | 1 | 41 | 132 | 24.45 | 5.49 | 5.27 | 1.12 | 0 | 0 | 0 | 0 | 0 | 0 |
| 21 | 1 | 0 | 42 | 112 | 22.10 | 6.20 | 4.11 | 0.67 | 0 | 0 | 0 | 0 | 0 | 0 |
| 22 | 2 | 1 | 53 | 92 | 21.13 | 5.73 | 4.53 | 0.63 | 0 | 0 | 0 | 0 | 0 | 1 |
| 23 | 1 | 0 | 30 | 127 | 23.34 | 5.00 | 3.15 | 1.16 | 0 | 0 | 0 | 0 | 0 | 0 |
| 24 | 1 | 0 | 83 | 104 | 15.63 | 6.83 | 3.54 | 0.77 | 0 | 0 | 0 | 0 | 0 | 0 |
| 25 | 1 | 1 | 39 | 112 | 28.40 | 5.80 | 2.92 | 0.56 | 0 | 0 | 0 | 0 | 0 | 0 |
| 26 | 1 | 0 | 64 | 114 | 19.98 | 5.49 | 4.37 | 0.91 | 0 | 0 | 0 | 0 | 0 | 0 |
| 27 | 1 | 0 | 27 | 106 | 18.11 | 4.93 | 5.10 | 0.63 | 0 | 0 | 0 | 0 | 0 | 0 |
| 28 | 1 | 0 | 67 | 128 | 22.04 | 5.68 | 3.69 | 0.57 | 0 | 0 | 0 | 0 | 0 | 0 |
| 29 | 1 | 0 | 55 | 112 | 28.58 | 5.05 | 4.55 | 0.88 | 0 | 0 | 0 | 0 | 0 | 0 |
| 30 | 2 | 1 | 73 | 132 | 18.73 | 7.15 | 2.08 | 0.56 | 0 | 0 | 0 | 0 | 0 | 0 |
| 31 | 1 | 0 | 49 | 133 | 24.40 | 4.96 | 3.79 | 0.79 | 0 | 0 | 0 | 0 | 0 | 0 |
| 32 | 2 | 1 | 89 | 117 | 23.31 | 5.36 | 4.49 | 0.65 | 1 | 0 | 0 | 0 | 0 | 0 |
| 33 | 2 | 1 | 53 | 143 | 23.14 | 4.38 | 4.36 | 0.70 | 0 | 0 | 0 | 0 | 0 | 0 |
| 34 | 1 | 0 | 50 | 121 | 30.78 | 6.38 | 4.22 | 1.08 | 0 | 0 | 0 | 0 | 0 | 0 |
| 35 | 5 | 0 | 71 | 163 | 30.44 | 5.86 | 4.14 | 1.38 | 0 | 0 | 0 | 1 | 0 | 1 |
| 36 | 1 | 0 | 88 | 126 | 22.89 | 4.75 | 3.70 | 0.57 | 0 | 0 | 0 | 0 | 0 | 0 |
| 37 | 1 | 1 | 32 | 137 | 21.85 | 5.34 | 5.36 | 1.19 | 0 | 0 | 0 | 0 | 0 | 0 |
| 38 | 1 | 0 | 64 | 96 | 16.02 | 6.09 | 3.09 | 0.67 | 0 | 0 | 0 | 0 | 0 | 0 |
| 39 | 1 | 0 | 40 | 122 | 23.44 | 5.15 | 4.98 | 0.83 | 0 | 0 | 0 | 0 | 0 | 0 |
| 40 | 1 | 1 | 54 | 108 | 21.79 | 5.49 | 4.63 | 1.08 | 0 | 0 | 0 | 0 | 0 | 0 |
| 41 | 1 | 0 | 57 | 116 | 24.96 | 4.90 | 3.89 | 0.73 | 0 | 0 | 0 | 0 | 0 | 0 |
| 42 | 2 | 0 | 55 | 124 | 31.56 | 4.67 | 4.14 | 0.85 | 1 | 0 | 0 | 0 | 0 | 0 |
| 43 | 2 | 1 | 51 | 137 | 25.95 | 4.88 | 6.48 | 0.99 | 0 | 0 | 0 | 0 | 0 | 0 |
| 44 | 3 | 1 | 62 | 104 | 28.63 | 4.48 | 4.39 | 0.77 | 1 | 0 | 0 | 0 | 1 | 0 |
| 45 | 4 | 0 | 57 | 143 | 25.51 | 4.52 | 2.52 | 1.11 | 1 | 0 | 0 | 0 | 0 | 0 |
| 46 | 1 | 1 | 42 | 133 | 20.96 | 5.93 | 4.84 | 1.58 | 0 | 0 | 0 | 0 | 0 | 0 |
| 47 | 2 | 1 | 51 | 109 | 27.07 | 5.56 | 3.05 | 0.90 | 0 | 0 | 0 | 0 | 1 | 0 |
| 48 | 1 | 0 | 22 | 126 | 20.32 | 4.66 | 4.41 | 1.15 | 0 | 0 | 0 | 0 | 0 | 0 |
| 49 | 1 | 1 | 78 | 130 | 24.80 | 5.80 | 4.49 | 0.89 | 0 | 0 | 0 | 0 | 0 | 0 |
| 50 | 2 | 1 | 63 | 101 | 22.09 | 7.91 | 2.99 | 1.53 | 1 | 0 | 0 | 0 | 0 | 0 |
| 51 | 3 | 1 | 54 | 134 | 23.99 | 4.83 | 6.15 | 1.40 | 0 | 0 | 0 | 0 | 0 | 1 |
| 52 | 5 | 1 | 68 | 165 | 25.71 | 14.95 | 2.61 | 0.82 | 0 | 0 | 0 | 0 | 1 | 0 |
| 53 | 3 | 1 | 58 | 129 | 25.07 | 5.92 | 5.28 | 1.20 | 0 | 0 | 0 | 0 | 0 | 1 |
| 54 | 2 | 1 | 54 | 113 | 21.09 | 4.54 | 4.37 | 0.94 | 0 | 0 | 0 | 0 | 0 | 1 |
| 55 | 4 | 0 | 58 | 148 | 26.04 | 5.18 | 6.25 | 0.80 | 1 | 0 | 0 | 0 | 0 | 0 |
| 56 | 4 | 1 | 46 | 150 | 25.25 | 6.34 | 2.99 | 0.97 | 1 | 0 | 0 | 0 | 0 | 0 |
| 57 | 2 | 0 | 78 | 117 | 25.21 | 4.59 | 3.67 | 1.23 | 1 | 0 | 0 | 0 | 0 | 0 |
| 58 | 4 | 1 | 54 | 145 | 28.72 | 4.60 | 6.08 | 1.09 | 1 | 0 | 0 | 0 | 0 | 0 |
| 59 | 3 | 1 | 66 | 114 | 22.50 | 4.61 | 3.08 | 1.56 | 1 | 0 | 0 | 0 | 0 | 1 |
| 60 | 5 | 1 | 80 | 140 | 20.28 | 7.37 | 2.78 | 1.08 | 1 | 0 | 0 | 1 | 0 | 0 |
| 61 | 3 | 1 | 61 | 138 | 23.05 | 4.35 | 2.25 | 0.88 | 1 | 0 | 0 | 0 | 0 | 0 |
| 62 | 5 | 1 | 51 | 150 | 27.07 | 5.56 | 3.05 | 0.90 | 1 | 0 | 0 | 0 | 1 | 0 |
| 63 | 4 | 1 | 93 | 147 | 22.49 | 4.41 | 4.88 | 1.09 | 1 | 0 | 0 | 0 | 0 | 0 |
| 64 | 3 | 1 | 75 | 108 | 21.48 | 5.99 | 4.19 | 1.07 | 1 | 1 | 0 | 0 | 0 | 0 |
| 65 | 2 | 0 | 86 | 120 | 22.03 | 5.08 | 3.24 | 1.02 | 1 | 0 | 0 | 0 | 0 | 0 |
| 66 | 5 | 0 | 63 | 124 | 20.44 | 4.86 | 4.09 | 0.75 | 0 | 0 | 0 | 0 | 3 | 0 |
| 67 | 1 | 1 | 41 | 118 | 22.13 | 5.65 | 5.97 | 0.61 | 0 | 0 | 0 | 0 | 0 | 0 |
| 68 | 4 | 1 | 31 | 145 | 21.72 | 11.20 | 6.35 | 1.15 | 0 | 0 | 0 | 0 | 1 | 0 |
| 69 | 3 | 1 | 59 | 124 | 17.67 | 6.53 | 4.38 | 0.85 | 0 | 0 | 0 | 1 | 0 | 0 |
| 70 | 1 | 1 | 73 | 123 | 21.88 | 4.47 | 2.84 | 0.89 | 0 | 0 | 0 | 0 | 0 | 0 |
| 71 | 2 | 1 | 38 | 148 | 23.26 | 5.95 | 2.76 | 0.94 | 0 | 0 | 0 | 0 | 0 | 0 |
| 72 | 2 | 0 | 79 | 114 | 27.34 | 5.75 | 2.16 | 1.55 | 1 | 0 | 0 | 0 | 0 | 0 |
| 73 | 3 | 0 | 72 | 124 | 21.43 | 6.47 | 5.27 | 0.60 | 0 | 0 | 0 | 0 | 0 | 1 |
| 74 | 1 | 1 | 38 | 138 | 27.90 | 6.46 | 4.98 | 1.15 | 0 | 0 | 0 | 0 | 0 | 0 |
| 75 | 4 | 0 | 73 | 144 | 24.25 | 4.89 | 5.17 | 0.95 | 0 | 0 | 0 | 0 | 0 | 1 |
| 76 | 3 | 1 | 69 | 103 | 18.78 | 11.50 | 5.56 | 0.78 | 1 | 0 | 0 | 1 | 0 | 0 |
| 77 | 3 | 1 | 43 | 131 | 27.04 | 5.56 | 3.11 | 0.83 | 1 | 0 | 0 | 0 | 0 | 0 |
| 78 | 4 | 0 | 27 | 171 | 24.84 | 6.21 | 4.84 | 0.92 | 1 | 0 | 0 | 0 | 0 | 0 |
| 79 | 3 | 0 | 64 | 133 | 23.78 | 5.07 | 4.14 | 0.94 | 1 | 0 | 0 | 0 | 0 | 0 |
| 80 | 2 | 1 | 54 | 124 | 21.93 | 6.77 | 4.74 | 0.97 | 1 | 0 | 0 | 0 | 0 | 0 |
| 81 | 2 | 0 | 53 | 106 | 28.36 | 5.26 | 4.64 | 1.20 | 1 | 0 | 0 | 0 | 0 | 0 |
| 82 | 1 | 1 | 59 | 122 | 21.05 | 5.63 | 4.90 | 0.67 | 0 | 0 | 0 | 0 | 0 | 0 |
| 83 | 3 | 1 | 59 | 135 | 24.22 | 6.34 | 3.09 | 0.83 | 1 | 0 | 0 | 0 | 0 | 0 |
| 84 | 3 | 0 | 56 | 140 | 20.03 | 8.80 | 4.23 | 0.80 | 1 | 0 | 0 | 0 | 0 | 0 |
| 85 | 2 | 1 | 62 | 131 | 19.72 | 6.12 | 4.33 | 0.56 | 0 | 0 | 0 | 0 | 0 | 0 |
| 86 | 4 | 0 | 66 | 156 | 25.81 | 5.65 | 4.19 | 0.95 | 1 | 0 | 0 | 0 | 0 | 0 |
| 87 | 2 | 0 | 61 | 160 | 28.58 | 5.27 | 5.69 | 0.64 | 0 | 0 | 0 | 0 | 0 | 0 |
| 88 | 3 | 1 | 62 | 131 | 23.31 | 5.85 | 3.50 | 0.74 | 1 | 0 | 0 | 0 | 0 | 0 |
| 89 | 3 | 1 | 77 | 136 | 29.72 | 6.10 | 3.24 | 1.48 | 1 | 0 | 0 | 0 | 0 | 0 |
| 90 | 3 | 1 | 71 | 138 | 23.99 | 4.65 | 4.74 | 0.87 | 1 | 0 | 0 | 0 | 0 | 0 |
| 91 | 5 | 0 | 66 | 135 | 25.71 | 6.71 | 2.29 | 0.90 | 1 | 0 | 0 | 0 | 1 | 0 |
| 92 | 4 | 0 | 74 | 145 | 23.01 | 5.15 | 3.73 | 0.95 | 0 | 0 | 0 | 0 | 0 | 1 |
| 93 | 3 | 1 | 63 | 127 | 26.28 | 7.68 | 4.26 | 1.01 | 1 | 0 | 0 | 0 | 0 | 0 |
| 94 | 2 | 1 | 32 | 141 | 22.40 | 5.74 | 5.96 | 1.22 | 0 | 0 | 0 | 0 | 0 | 0 |
| 95 | 4 | 1 | 50 | 226 | 26.12 | 5.61 | 2.44 | 0.53 | 0 | 0 | 0 | 0 | 0 | 1 |
| 96 | 3 | 0 | 58 | 138 | 30.04 | 5.63 | 3.70 | 0.93 | 1 | 0 | 0 | 0 | 0 | 0 |
| 97 | 5 | 1 | 48 | 173 | 25.46 | 7.46 | 4.28 | 0.65 | 1 | 0 | 0 | 0 | 0 | 1 |
| 98 | 3 | 0 | 34 | 150 | 23.98 | 5.53 | 4.39 | 0.61 | 1 | 0 | 0 | 0 | 0 | 0 |
| 99 | 4 | 0 | 71 | 148 | 21.07 | 5.44 | 3.52 | 0.60 | 0 | 0 | 0 | 0 | 1 | 0 |
| 100 | 5 | 1 | 39 | 155 | 28.96 | 12.01 | 4.33 | 1.24 | 1 | 0 | 0 | 1 | 0 | 0 |
| 101 | 4 | 1 | 76 | 153 | 20.20 | 4.18 | 2.68 | 1.28 | 0 | 0 | 0 | 0 | 1 | 0 |
| 102 | 5 | 1 | 65 | 146 | 25.23 | 6.14 | 3.55 | 0.78 | 1 | 0 | 0 | 1 | 0 | 0 |
| 103 | 3 | 1 | 52 | 134 | 23.89 | 5.48 | 5.45 | 1.23 | 1 | 0 | 0 | 0 | 0 | 0 |
| 104 | 4 | 0 | 62 | 160 | 28.89 | 5.32 | 5.37 | 0.65 | 1 | 0 | 0 | 0 | 0 | 0 |
| 105 | 4 | 0 | 78 | 102 | 20.00 | 8.11 | 3.54 | 0.56 | 1 | 0 | 0 | 1 | 0 | 1 |
| 106 | 2 | 0 | 55 | 124 | 31.56 | 4.67 | 4.14 | 0.85 | 1 | 0 | 0 | 0 | 0 | 0 |
| 107 | 4 | 0 | 70 | 128 | 26.67 | 8.16 | 3.89 | 0.81 | 1 | 0 | 0 | 0 | 0 | 1 |
| 108 | 3 | 1 | 62 | 136 | 25.34 | 5.87 | 2.76 | 0.95 | 1 | 0 | 0 | 0 | 0 | 0 |
| 109 | 4 | 1 | 63 | 157 | 25.09 | 5.65 | 3.95 | 1.04 | 1 | 0 | 0 | 0 | 0 | 0 |
| 110 | 3 | 1 | 68 | 114 | 23.74 | 5.29 | 2.56 | 0.64 | 1 | 1 | 0 | 0 | 0 | 0 |
| 111 | 4 | 1 | 61 | 136 | 27.08 | 10.60 | 4.66 | 1.08 | 1 | 0 | 0 | 0 | 0 | 0 |
| 112 | 3 | 1 | 71 | 96 | 24.45 | 7.79 | 4.16 | 0.69 | 1 | 0 | 0 | 0 | 1 | 0 |
| 113 | 2 | 1 | 70 | 118 | 25.88 | 5.90 | 3.81 | 1.15 | 0 | 0 | 0 | 0 | 1 | 0 |
| 114 | 5 | 0 | 63 | 147 | 30.04 | 4.86 | 3.46 | 0.76 | 1 | 0 | 0 | 0 | 0 | 1 |
| 115 | 3 | 1 | 70 | 129 | 25.84 | 5.33 | 4.41 | 1.02 | 1 | 0 | 0 | 0 | 0 | 0 |
| 116 | 3 | 0 | 51 | 120 | 32.05 | 4.23 | 2.66 | 0.73 | 1 | 1 | 0 | 0 | 0 | 0 |
| 117 | 2 | 1 | 63 | 108 | 26.12 | 6.86 | 5.13 | 1.24 | 1 | 0 | 0 | 0 | 0 | 0 |
| 118 | 1 | 0 | 35 | 99 | 16.80 | 4.14 | 3.46 | 0.73 | 0 | 0 | 0 | 0 | 0 | 0 |
| 119 | 4 | 0 | 60 | 122 | 20.20 | 5.46 | 4.62 | 0.92 | 1 | 0 | 0 | 0 | 1 | 0 |
| 120 | 3 | 1 | 63 | 125 | 21.09 | 4.58 | 3.57 | 1.28 | 1 | 0 | 0 | 0 | 0 | 0 |
| 121 | 2 | 1 | 68 | 101 | 22.28 | 5.06 | 3.39 | 1.09 | 1 | 0 | 0 | 0 | 0 | 0 |
| 122 | 5 | 0 | 77 | 126 | 24.97 | 6.30 | 4.64 | 0.89 | 1 | 0 | 0 | 1 | 0 | 1 |
| 123 | 4 | 1 | 68 | 140 | 24.22 | 5.74 | 4.03 | 0.98 | 1 | 0 | 0 | 1 | 0 | 0 |
| 124 | 3 | 0 | 81 | 93 | 20.93 | 7.00 | 4.47 | 0.70 | 1 | 0 | 0 | 0 | 0 | 1 |
| 125 | 3 | 0 | 86 | 134 | 28.25 | 5.13 | 3.91 | 1.06 | 1 | 0 | 0 | 0 | 0 | 0 |
| 126 | 3 | 1 | 42 | 134 | 23.56 | 8.70 | 4.47 | 0.68 | 1 | 0 | 0 | 0 | 0 | 0 |
| 127 | 3 | 1 | 68 | 139 | 19.97 | 4.47 | 2.07 | 0.68 | 1 | 0 | 0 | 0 | 0 | 0 |
| 128 | 3 | 1 | 56 | 124 | 19.49 | 5.00 | 4.84 | 0.58 | 0 | 0 | 0 | 0 | 0 | 1 |
| 129 | 5 | 0 | 72 | 160 | 28.91 | 5.67 | 4.10 | 0.89 | 1 | 0 | 0 | 1 | 0 | 0 |
| 130 | 2 | 1 | 85 | 104 | 22.94 | 7.71 | 5.30 | 1.14 | 1 | 0 | 0 | 0 | 0 | 0 |
| 131 | 4 | 0 | 77 | 133 | 25.42 | 10.98 | 3.04 | 0.89 | 1 | 0 | 0 | 0 | 0 | 0 |
| 132 | 1 | 1 | 31 | 135 | 24.49 | 5.75 | 5.26 | 1.04 | 0 | 0 | 0 | 0 | 0 | 0 |
| 133 | 5 | 1 | 78 | 157 | 18.22 | 5.99 | 4.77 | 0.92 | 1 | 0 | 1 | 0 | 0 | 0 |
| 134 | 3 | 1 | 77 | 129 | 25.95 | 7.15 | 2.71 | 1.17 | 0 | 0 | 0 | 0 | 0 | 1 |
| 135 | 5 | 1 | 58 | 126 | 31.71 | 5.43 | 3.35 | 1.06 | 1 | 0 | 0 | 1 | 1 | 0 |
| 136 | 2 | 0 | 52 | 145 | 24.22 | 5.83 | 5.60 | 1.26 | 0 | 0 | 0 | 0 | 0 | 0 |
| 137 | 4 | 1 | 26 | 170 | 25.79 | 4.16 | 3.10 | 1.24 | 1 | 0 | 0 | 0 | 0 | 0 |
| 138 | 4 | 1 | 51 | 143 | 30.09 | 5.47 | 3.70 | 1.52 | 1 | 0 | 0 | 0 | 0 | 0 |
| 139 | 4 | 1 | 64 | 153 | 25.10 | 4.70 | 3.25 | 1.26 | 1 | 0 | 0 | 0 | 0 | 0 |
| 140 | 4 | 1 | 57 | 151 | 28.65 | 5.27 | 4.44 | 1.61 | 1 | 0 | 0 | 0 | 0 | 0 |
| 141 | 4 | 0 | 87 | 124 | 17.48 | 4.53 | 2.68 | 1.04 | 1 | 1 | 0 | 0 | 0 | 0 |
| 142 | 3 | 0 | 65 | 98 | 20.40 | 5.86 | 2.49 | 1.11 | 1 | 0 | 0 | 1 | 0 | 0 |
| 143 | 4 | 0 | 58 | 132 | 29.30 | 6.28 | 4.76 | 0.93 | 0 | 1 | 0 | 0 | 1 | 0 |
| 144 | 5 | 1 | 75 | 170 | 30.55 | 8.15 | 3.29 | 1.28 | 1 | 1 | 0 | 0 | 1 | 1 |
| 145 | 5 | 0 | 60 | 164 | 26.04 | 4.34 | 2.92 | 0.57 | 1 | 0 | 0 | 0 | 1 | 0 |
| 146 | 3 | 1 | 64 | 136 | 27.43 | 5.33 | 4.88 | 1.07 | 1 | 0 | 0 | 0 | 0 | 0 |
| 147 | 3 | 1 | 64 | 125 | 28.58 | 3.30 | 3.14 | 1.45 | 1 | 0 | 0 | 0 | 0 | 0 |
| 148 | 4 | 0 | 65 | 134 | 21.69 | 4.56 | 3.52 | 0.59 | 0 | 0 | 1 | 1 | 0 | 0 |
| 149 | 5 | 1 | 52 | 173 | 23.83 | 6.01 | 4.96 | 1.28 | 1 | 0 | 0 | 0 | 0 | 1 |
| 150 | 4 | 1 | 57 | 125 | 24.80 | 9.16 | 5.60 | 0.97 | 1 | 1 | 0 | 0 | 0 | 0 |
| 151 | 3 | 1 | 70 | 127 | 25.95 | 6.20 | 5.74 | 1.30 | 1 | 0 | 0 | 0 | 0 | 0 |
| 152 | 5 | 0 | 75 | 135 | 27.56 | 5.42 | 6.23 | 0.93 | 1 | 0 | 0 | 0 | 0 | 1 |
| 153 | 3 | 1 | 80 | 140 | 17.15 | 5.57 | 3.19 | 0.71 | 0 | 0 | 0 | 0 | 1 | 0 |
| 154 | 4 | 0 | 53 | 143 | 21.95 | 4.30 | 4.76 | 0.67 | 1 | 0 | 0 | 0 | 0 | 0 |
| 155 | 4 | 1 | 65 | 142 | 22.09 | 8.18 | 5.35 | 1.45 | 0 | 0 | 0 | 0 | 1 | 0 |
| 156 | 5 | 0 | 65 | 168 | 30.93 | 6.04 | 5.54 | 1.55 | 1 | 0 | 0 | 0 | 0 | 0 |
| 157 | 2 | 1 | 71 | 105 | 22.66 | 5.10 | 3.58 | 1.09 | 0 | 0 | 0 | 0 | 0 | 1 |
| 158 | 5 | 1 | 80 | 142 | 21.63 | 13.65 | 4.47 | 0.81 | 1 | 1 | 0 | 0 | 0 | 0 |
| 159 | 4 | 1 | 26 | 144 | 28.41 | 4.16 | 4.15 | 0.80 | 1 | 0 | 0 | 0 | 1 | 0 |
| 160 | 4 | 0 | 58 | 145 | 21.88 | 6.56 | 5.64 | 0.61 | 1 | 0 | 0 | 0 | 0 | 0 |
| 161 | 2 | 0 | 59 | 147 | 31.22 | 8.25 | 5.26 | 1.52 | 0 | 0 | 0 | 0 | 0 | 0 |
| 162 | 3 | 1 | 57 | 113 | 21.26 | 8.36 | 3.57 | 0.92 | 0 | 0 | 0 | 0 | 1 | 1 |
| 163 | 2 | 0 | 56 | 142 | 24.77 | 7.14 | 4.61 | 0.61 | 0 | 0 | 0 | 0 | 0 | 0 |
| 164 | 2 | 1 | 78 | 89 | 20.12 | 7.03 | 3.30 | 1.07 | 1 | 0 | 0 | 0 | 0 | 0 |
| 165 | 5 | 1 | 71 | 123 | 22.27 | 5.10 | 3.58 | 1.09 | 1 | 1 | 0 | 1 | 1 | 0 |
| 166 | 5 | 1 | 79 | 167 | 24.80 | 6.00 | 2.98 | 0.56 | 1 | 0 | 0 | 0 | 0 | 0 |
| 167 | 2 | 1 | 50 | 125 | 26.12 | 5.61 | 2.44 | 0.53 | 1 | 0 | 0 | 0 | 0 | 0 |
| 168 | 2 | 1 | 48 | 150 | 26.78 | 5.43 | 5.13 | 1.04 | 0 | 0 | 0 | 0 | 0 | 0 |
| 169 | 5 | 0 | 77 | 174 | 26.67 | 8.36 | 3.57 | 0.94 | 1 | 0 | 0 | 0 | 0 | 0 |
| 170 | 2 | 0 | 40 | 124 | 29.78 | 4.85 | 5.24 | 1.45 | 1 | 0 | 0 | 0 | 0 | 0 |
| 171 | 2 | 0 | 81 | 93 | 20.93 | 7.00 | 4.47 | 0.82 | 1 | 0 | 0 | 0 | 0 | 0 |
| 172 | 4 | 0 | 72 | 129 | 23.88 | 5.47 | 4.60 | 0.56 | 1 | 1 | 0 | 0 | 0 | 0 |
| 173 | 4 | 0 | 62 | 132 | 23.23 | 6.35 | 4.18 | 0.90 | 1 | 0 | 0 | 0 | 0 | 1 |
| 174 | 5 | 0 | 63 | 170 | 21.31 | 6.45 | 6.51 | 0.95 | 0 | 0 | 0 | 0 | 1 | 0 |
| 175 | 4 | 1 | 87 | 123 | 16.53 | 3.94 | 3.68 | 0.70 | 1 | 1 | 0 | 0 | 0 | 0 |
| 176 | 5 | 0 | 56 | 180 | 19.95 | 8.42 | 4.89 | 0.58 | 1 | 0 | 0 | 1 | 0 | 0 |
| 177 | 5 | 0 | 77 | 169 | 19.60 | 7.53 | 4.33 | 0.64 | 1 | 0 | 0 | 0 | 0 | 0 |
| 178 | 5 | 0 | 65 | 162 | 21.69 | 4.56 | 3.52 | 0.59 | 1 | 0 | 0 | 1 | 0 | 0 |
| 179 | 3 | 0 | 70 | 117 | 24.03 | 5.39 | 6.07 | 1.39 | 1 | 1 | 0 | 0 | 0 | 0 |
| 180 | 5 | 1 | 72 | 142 | 24.84 | 5.33 | 4.89 | 1.09 | 1 | 0 | 0 | 0 | 0 | 1 |
| 181 | 4 | 1 | 58 | 149 | 23.80 | 5.57 | 4.65 | 1.16 | 0 | 0 | 0 | 1 | 0 | 0 |
| 182 | 2 | 0 | 65 | 142 | 28.32 | 4.69 | 4.45 | 0.72 | 0 | 0 | 0 |  | 0 | 0 |
| 183 | 3 | 1 | 53 | 135 | 24.49 | 4.89 | 3.87 | 0.70 | 0 | 0 | 0 | 0 | 0 | 1 |
| 184 | 5 | 1 | 56 | 170 | 26.45 | 5.37 | 5.53 | 0.98 | 0 | 0 | 0 | 0 | 1 | 0 |
| 185 | 4 | 1 | 42 | 170 | 33.63 | 4.85 | 3.61 | 1.46 | 0 | 0 | 1 | 0 | 0 | 0 |
| 186 | 5 | 1 | 75 | 178 | 24.34 | 6.25 | 2.99 | 1.08 | 1 | 1 | 0 | 0 | 0 | 0 |
| 187 | 5 | 1 | 58 | 154 | 27.68 | 5.40 | 3.64 | 1.16 | 0 | 0 | 0 | 0 | 1 | 1 |
| 188 | 5 | 1 | 64 | 160 | 25.95 | 5.32 | 5.73 | 0.59 | 0 | 0 | 0 | 0 | 1 | 1 |
| 189 | 5 | 0 | 52 | 174 | 28.72 | 6.53 | 6.27 | 0.57 | 0 | 0 | 0 | 0 | 1 | 0 |
| 190 | 2 | 1 | 49 | 122 | 20.76 | 5.13 | 3.11 | 0.59 | 1 | 0 | 0 | 0 | 0 | 0 |
| 191 | 5 | 1 | 65 | 168 | 24.22 | 6.69 | 4.83 | 0.77 | 1 | 0 | 0 | 0 | 0 | 1 |
| 192 | 2 | 1 | 50 | 147 | 26.12 | 5.61 | 2.44 | 0.53 | 0 | 0 | 0 | 0 | 0 | 0 |
| 193 | 5 | 1 | 85 | 143 | 27.34 | 13.20 | 5.16 | 0.64 | 1 | 1 | 0 | 0 | 1 | 0 |
| 194 | 5 | 1 | 49 | 141 | 29.00 | 8.33 | 5.44 | 0.52 | 1 | 0 | 0 | 1 | 0 | 0 |
| 195 | 5 | 1 | 67 | 131 | 24.91 | 7.49 | 3.76 | 0.88 | 1 | 1 | 0 | 0 | 0 | 0 |
| 196 | 4 | 0 | 65 | 117 | 18.73 | 4.76 | 5.77 | 1.11 | 1 | 1 | 0 | 0 | 1 | 0 |
| 197 | 5 | 0 | 58 | 158 | 23.83 | 5.15 | 6.29 | 1.12 | 1 | 0 | 0 | 0 | 0 | 1 |
| 198 | 4 | 1 | 74 | 139 | 23.14 | 5.35 | 5.16 | 1.16 | 1 | 0 | 0 | 0 | 0 | 1 |
| 199 | 5 | 1 | 39 | 176 | 28.34 | 6.34 | 5.57 | 0.56 | 0 | 0 | 1 | 1 | 1 | 1 |
| 200 | 5 | 1 | 84 | 150 | 23.40 | 4.74 | 3.14 | 1.15 | 1 | 1 | 0 | 0 | 1 | 0 |

**Tab. S17** Clinical analysis results and information of verification group

| Number | Clinical Diagnosis | Gender | Age | Systolic blood pressure | BMI | Glucose | Total Chol | Lactate | History of hypertension | History of Heart attack | History of Cardiac insufficiency | Cerebral infarction history | History of diabetes | Smoking history |
| --- | --- | --- | --- | --- | --- | --- | --- | --- | --- | --- | --- | --- | --- | --- |
| 1 | 1 | 0 | 29 | 110 | 25.22 | 5.11 | 5.45 | 0.62 | 0 | 0 | 0 | 0 | 0 | 0 |
| 2 | 1 | 1 | 31 | 116 | 26.37 | 5.51 | 3.66 | 0.99 | 0 | 0 | 0 | 0 | 0 | 0 |
| 3 | 1 | 1 | 60 | 129 | 24.80 | 4.74 | 5.11 | 0.98 | 0 | 0 | 0 | 0 | 0 | 0 |
| 4 | 1 | 1 | 52 | 122 | 23.79 | 6.85 | 2.91 | 0.73 | 0 | 0 | 0 | 0 | 0 | 0 |
| 5 | 1 | 0 | 34 | 103 | 21.03 | 4.54 | 3.02 | 1.05 | 0 | 0 | 0 | 0 | 0 | 0 |
| 6 | 1 | 1 | 65 | 112 | 24.15 | 7.98 | 3.26 | 1.18 | 0 | 0 | 0 | 0 | 0 | 0 |
| 7 | 1 | 1 | 40 | 118 | 20.76 | 4.62 | 4.12 | 1.24 | 0 | 0 | 0 | 0 | 0 | 0 |
| 8 | 1 | 1 | 53 | 107 | 24.64 | 4.82 | 3.31 | 1.00 | 0 | 0 | 0 | 0 | 0 | 0 |
| 9 | 1 | 0 | 57 | 130 | 23.67 | 4.22 | 4.76 | 0.90 | 0 | 0 | 0 | 0 | 0 | 0 |
| 10 | 1 | 0 | 46 | 111 | 24.01 | 4.78 | 5.65 | 0.65 | 0 | 0 | 0 | 0 | 0 | 0 |
| 11 | 1 | 1 | 68 | 105 | 23.83 | 6.31 | 4.44 | 1.04 | 0 | 0 | 0 | 0 | 0 | 0 |
| 12 | 2 | 1 | 54 | 134 | 23.02 | 5.10 | 5.75 | 1.06 | 0 | 0 | 0 | 0 | 0 | 0 |
| 13 | 2 | 1 | 44 | 104 | 26.23 | 4.61 | 5.28 | 1.09 | 0 | 1 | 0 | 0 | 0 | 0 |
| 14 | 1 | 0 | 62 | 116 | 20.90 | 5.00 | 5.52 | 0.76 | 0 | 0 | 0 | 0 | 0 | 0 |
| 15 | 1 | 0 | 51 | 102 | 21.50 | 4.54 | 5.71 | 0.64 | 0 | 0 | 0 | 0 | 0 | 0 |
| 16 | 2 | 0 | 55 | 131 | 23.31 | 5.69 | 3.30 | 0.57 | 0 | 0 | 0 | 0 | 0 | 0 |
| 17 | 1 | 0 | 32 | 128 | 23.64 | 4.78 | 3.66 | 0.56 | 0 | 0 | 0 | 0 | 0 | 0 |
| 18 | 2 | 0 | 50 | 132 | 23.75 | 4.57 | 5.24 | 0.62 | 0 | 0 | 0 | 0 | 0 | 0 |
| 19 | 1 | 0 | 63 | 116 | 20.96 | 4.70 | 4.71 | 0.78 | 0 | 0 | 0 | 0 | 0 | 0 |
| 20 | 2 | 1 | 41 | 132 | 24.45 | 5.49 | 5.27 | 1.12 | 0 | 0 | 0 | 0 | 0 | 0 |
| 21 | 1 | 0 | 42 | 112 | 22.10 | 6.20 | 4.11 | 0.67 | 0 | 0 | 0 | 0 | 0 | 0 |
| 22 | 2 | 1 | 53 | 92 | 21.13 | 5.73 | 4.53 | 0.63 | 0 | 0 | 0 | 0 | 0 | 1 |
| 23 | 1 | 0 | 30 | 127 | 23.34 | 5.00 | 3.15 | 1.16 | 0 | 0 | 0 | 0 | 0 | 0 |
| 24 | 1 | 0 | 83 | 104 | 15.63 | 6.83 | 3.54 | 0.77 | 0 | 0 | 0 | 0 | 0 | 0 |
| 25 | 1 | 1 | 39 | 112 | 28.40 | 5.80 | 2.92 | 0.56 | 0 | 0 | 0 | 0 | 0 | 0 |
| 26 | 1 | 0 | 64 | 114 | 19.98 | 5.49 | 4.37 | 0.91 | 0 | 0 | 0 | 0 | 0 | 0 |
| 27 | 1 | 0 | 27 | 106 | 18.11 | 4.93 | 5.10 | 0.63 | 0 | 0 | 0 | 0 | 0 | 0 |
| 28 | 1 | 0 | 67 | 128 | 22.04 | 5.68 | 3.69 | 0.57 | 0 | 0 | 0 | 0 | 0 | 0 |
| 29 | 1 | 0 | 55 | 112 | 28.58 | 5.05 | 4.55 | 0.88 | 0 | 0 | 0 | 0 | 0 | 0 |
| 30 | 2 | 1 | 73 | 132 | 18.73 | 7.15 | 2.08 | 0.56 | 0 | 0 | 0 | 0 | 0 | 0 |
| 31 | 1 | 0 | 49 | 133 | 24.40 | 4.96 | 3.79 | 0.79 | 0 | 0 | 0 | 0 | 0 | 0 |
| 32 | 2 | 1 | 89 | 117 | 23.31 | 5.36 | 4.49 | 0.65 | 1 | 0 | 0 | 0 | 0 | 0 |
| 33 | 2 | 1 | 53 | 143 | 23.14 | 4.38 | 4.36 | 0.70 | 0 | 0 | 0 | 0 | 0 | 0 |
| 34 | 1 | 0 | 50 | 121 | 30.78 | 6.38 | 4.22 | 1.08 | 0 | 0 | 0 | 0 | 0 | 0 |
| 35 | 5 | 0 | 71 | 163 | 30.44 | 5.86 | 4.14 | 1.38 | 0 | 0 | 0 | 1 | 0 | 1 |
| 36 | 1 | 0 | 88 | 126 | 22.89 | 4.75 | 3.70 | 0.57 | 0 | 0 | 0 | 0 | 0 | 0 |
| 37 | 1 | 1 | 32 | 137 | 21.85 | 5.34 | 5.36 | 1.19 | 0 | 0 | 0 | 0 | 0 | 0 |
| 38 | 1 | 0 | 64 | 96 | 16.02 | 6.09 | 3.09 | 0.67 | 0 | 0 | 0 | 0 | 0 | 0 |
| 39 | 1 | 0 | 40 | 122 | 23.44 | 5.15 | 4.98 | 0.83 | 0 | 0 | 0 | 0 | 0 | 0 |
| 40 | 1 | 1 | 54 | 108 | 21.79 | 5.49 | 4.63 | 1.08 | 0 | 0 | 0 | 0 | 0 | 0 |
| 41 | 1 | 0 | 57 | 116 | 24.96 | 4.90 | 3.89 | 0.73 | 0 | 0 | 0 | 0 | 0 | 0 |
| 42 | 2 | 0 | 55 | 124 | 31.56 | 4.67 | 4.14 | 0.85 | 1 | 0 | 0 | 0 | 0 | 0 |

3 Language Editing Certificate


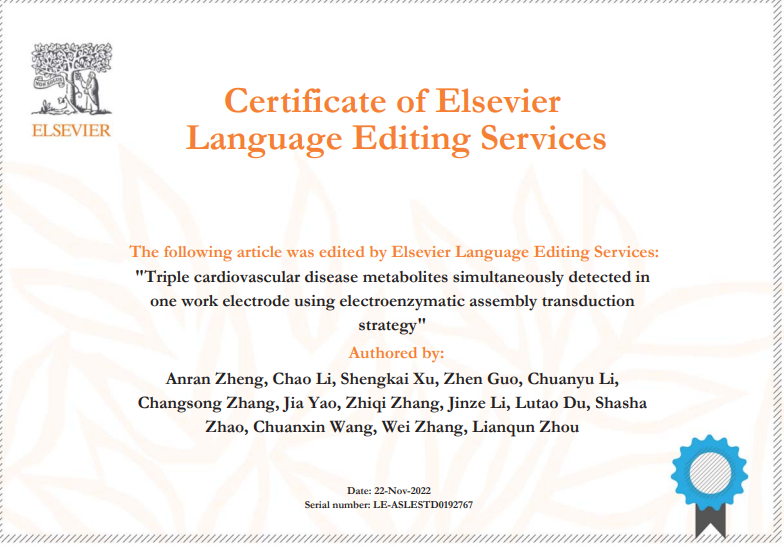


4 Reference

[1] W. Deng, S. Tang, X. Zhou, Y. Liu, S. Liu, J. Luo, Honeycomb-like structure-tunable chitosan-based porous carbon microspheres for methylene blue efficient removal, Carbohydr Polym 247 (2020) 116736.

[2] A. Esokkiya, S. Sudalaimani, K. Sanjeev Kumar, P. Sampathkumar, C. Suresh, K. Giribabu, Poly(methylene blue)-Based Electrochemical Platform for Label-Free Sensing of Acrylamide, ACS Omega 6(14) (2021) 9528-9536.

[3] S.R. Chinnadayyala, A. Kakoti, M. Santhosh, P. Goswami, A novel amperometric alcohol biosensor developed in a 3rd generation bioelectrode platform using peroxidase coupled ferrocene activated alcohol oxidase as biorecognition system, Biosens Bioelectron 55 (2014) 120-6.

[4] R. Pylypchuk, S. Wells, A. Kerr, K. Poppe, T. Riddell, M. Harwood, D. Exeter, S. Mehta, C. Grey, B.P. Wu, P. Metcalf, J. Warren, J. Harrison, R. Marshall, R. Jackson, Cardiovascular disease risk prediction equations in 400 000 primary care patients in New Zealand: a derivation and validation study, The Lancet 391(10133) (2018) 1897-1907.

[5] S.S. Mahmood, D. Levy, R.S. Vasan, T.J. Wang, The Framingham Heart Study and the epidemiology of cardiovascular disease: a historical perspective, The Lancet 383(9921) (2014) 999-1008.

[6] S.R. Yurista, C.R. Chong, J.J. Badimon, D.P. Kelly, R.A. de Boer, B.D. Westenbrink, Therapeutic Potential of Ketone Bodies for Patients With Cardiovascular Disease: JACC State-of-the-Art Review, J Am Coll Cardiol 77(13) (2021) 1660-1669.

[7] A. Dubatovka, J.M. Buhmann, Automatic Detection of Atrial Fibrillation from Single-Lead ECG Using Deep Learning of the Cardiac Cycle, BME Frontiers 2022 (2022) 1-12.

[8] J. Xu, Z. Wei, X. Wang, X. Li, W. Wang, The risk of cardiovascular and cerebrovascular disease in overlap syndrome: a meta-analysis, J Clin Sleep Med 16(7) (2020) 1199-1207.

[9] D.M. Lloyd-Jones, Cardiovascular risk prediction: basic concepts, current status, and future directions, Circulation 121(15) (2010) 1768-77.
